# Behaviour change techniques targeting both diet and physical activity in type 2 diabetes: A systematic review and meta-analysis

**DOI:** 10.1186/s12966-016-0436-0

**Published:** 2017-02-08

**Authors:** Kevin A. Cradock, Gearóid ÓLaighin, Francis M. Finucane, Heather L. Gainforth, Leo R. Quinlan, Kathleen A. Martin Ginis

**Affiliations:** 1 Physiology, School of Medicine, NUI Galway, University Road, Galway, Ireland; 2 Electrical & Electronic Engineering, School of Engineering & Informatics, NUI Galway, University Road, Galway, Ireland; 3 National Centre for Biomedical Engineering Science, NUI Galway, University Road, Galway, Ireland; 4 Bariatric Medicine Service, Galway Diabetes Research Centre, HRB Clinical Research Facility, Galway, Ireland; 5 School of Health and Exercise Sciences, Faculty of Health and Social Development, The University of British Columbia, ART 129– 1147, Research Road, Kelowna, BC V1V 1 V7 Canada; 6 School of Health and Exercise Sciences, Faculty of Health and Social Development, The University of British Columbia, ART 129-1147 Research Road, Kelowna, BC V1V 1 V7 Canada

**Keywords:** Behaviour change techniques, Diet, Physical activity, Type 2 diabetes, HbA_1c_, Systematic review, Meta-analysis

## Abstract

**Background:**

Changing diet and physical activity behaviour is one of the cornerstones of type 2 diabetes treatment, but changing behaviour is challenging**.** The objective of this study was to identify behaviour change techniques (BCTs) and intervention features of dietary and physical activity interventions for patients with type 2 diabetes that are associated with changes in HbA_1c_ and body weight.

**Methods:**

We performed a systematic review of papers published between 1975–2015 describing randomised controlled trials (RCTs) that focused exclusively on both diet and physical activity. The constituent BCTs, intervention features and methodological rigour of these interventions were evaluated. Changes in HbA_1c_ and body weight were meta-analysed and examined in relation to use of BCTs.

**Results:**

Thirteen RCTs were identified. Meta-analyses revealed reductions in HbA_1c_ at 3, 6, 12 and 24 months of -1.11 % (12 mmol/mol), -0.67 % (7 mmol/mol), -0.28 % (3 mmol/mol) and -0.26 % (2 mmol/mol) with an overall reduction of -0.53 % (6 mmol/mol [95 % CI -0.74 to -0.32, *P* < 0.00001]) in intervention groups compared to control groups. Meta-analyses also showed a reduction in body weight of -2.7 kg, -3.64 kg, -3.77 kg and -3.18 kg at 3, 6, 12 and 24 months, overall reduction was -3.73 kg (95 % CI -6.09 to -1.37 kg, *P* = 0.002).

Four of 46 BCTs identified were associated with >0.3 % reduction in HbA_1c_: ‘instruction on how to perform a behaviour’, ‘behavioural practice/rehearsal’, ‘demonstration of the behaviour’ and ‘action planning’, as were intervention features ‘supervised physical activity’, ‘group sessions’, ‘contact with an exercise physiologist’, ‘contact with an exercise physiologist and a dietitian’, ‘baseline HbA_1c_ >8 %’ and interventions of greater frequency and intensity.

**Conclusions:**

Diet and physical activity interventions achieved clinically significant reductions in HbA_1c_ at three and six months, but not at 12 and 24 months. Specific BCTs and intervention features identified may inform more effective structured lifestyle intervention treatment strategies for type 2 diabetes.

**Electronic supplementary material:**

The online version of this article (doi:10.1186/s12966-016-0436-0) contains supplementary material, which is available to authorized users.

## Background

Type 2 diabetes is one of the fastest growing and largest global health burdens. In 2015, there were 415 million people with diabetes worldwide (91 % of which were type 2 diabetes) with figures expected to rise to 642 million by the year 2040, [1] which easily surpasses earlier predictions of 366 million by 2030 [2]. A 2010 global analysis of mortality reported that 1.3 million deaths worldwide were due to diabetes that year, twice as many as in 1990 [3].

Type 2 diabetes is diagnosed based on a fasting plasma glucose (FPG ≥126 mg/dL [7 mmol/L]) or the two hour plasma glucose value following a 75 g oral glucose tolerance test (>200 mg/DL [11.0 mmol/L]) or having a HbA_1c_ of ≥ 6.5 % according to the American Diabetes Association (ADA) [4]. Glycosylated haemoglobin A_1c_ (HbA_1c_ haemoglobin to which glucose is bound, is tested to determine average blood glucose level over the past two to three months) [1] is widely regarded as an accurate measurement for diabetes assessment and the ADA recommend that HbA_1c_ testing be performed on all patients with diabetes at initial diagnosis and as part of continuing treatment [4]. HbA_1c_ reduction of 0.5 % (6 mmol/mol) is regarded as clinically significant [5], while other authors suggest 0.3 % (4 mmol/mol) [6, 7] or 0.33 % (4 mmol/mol) [8]. HbA_1c_ was selected as the primary outcome for this review as it represents the most widely used measure of type 2 diabetes control and treatment efficacy.

Type 2 diabetes is a mulifactorial lifestyle disease, linked to dietary habits and sedentary behaviour [9]. The ADA included ‘support patient behavioural change’ as one of their three key objectives for improving diabetes care and stated that ‘lifestyle changes of increasing physical activity, eating a healthy diet, cessation of smoking, weight loss and coping strategies’ was one of their key diabetes treatment foci [4]. Importantly, all three ADA treatment foci revolve around changing patients’ behaviour.

RCTs and epidemiological data have shown that type 2 diabetes can be prevented. However, changing diet and lifestyle behaviour requires change at an individual, environmental, social, and policy level [10]. Previous authors have identified as key research recommendations the need to investigate the effects of multiple behaviour changes in people who have been diagnosed with type 2 diabetes [11] and multiple BCT use associated with clinically significant changes in HbA_1c_ [7].

Precise specification of the active ingredients (BCTs) and intervention features of diet and physical activity interventions in type 2 diabetes will help build cumulative evidence towards delivering effective replicable interventions. Behaviour change technqiues (BCTs) have been identified in previous similar studies of diet and/or physical activity in type 2 diabetes [7, 12] and other subjects [13–16]. Previously identified BCTs associated with success in changing diet and/or physical activity behaviour include: ‘instruction on how to perform a behaviour’, ‘behavioural practice/rehearsal’, ‘demonstration of the behaviour’, ‘action planning’, ‘problem solving’, ‘feedback on behaviour’, ‘self-monitoring of behaviour’, ‘goal setting’, ‘goal review’, ‘social support’, ‘prompt practice’, ‘use of follow up prompts’, and ‘prompting generalisation of a target behaviour’ [7, 12–15, 17, 18].

However, to our knowledge, there has been no systematic review and meta-analysis identifying the behaviour change techniques (BCTs) associated with greatest improvements in HbA_1c_ in interventions combining diet and physical activity in type 2 diabetes treatment. We sought to identify which BCTs exclusively change only the behaviours of diet and physical activity. Interventions containing multiple behaviours or additional behaviours were not included in this review. Behaviour change has contributed to the morbidity and mortality associated with type 2 diabetes [19] but might also contribute to the solution [20]. However the effectiveness of behaviour change interventions varies considerably and their mechanisms are not fully understood [20]. The overall effects of diet and physical activity behavioural interventions in maintaining weight loss are moderate and future research on increasing effectiveness of interventions is required [21].

The primary objective of this study was to identify BCTs and intervention features which reduced HbA_1c_. A secondary objective was to identify the frequency of use of BCTs in included studies. A third objective was to describe changes in HbA_1c_ and weight at different time points.

## Methods

A PRISMA (Preferred Reporting Items for Systematic Reviews and Meta-Analyses) checklist was created and PRISMA review guidelines were followed [22] (Additional file 1: 1.1).

### Inclusion criteria

(i) Randomised controlled trials (RCTs) of any duration with a dietary AND physical activity intervention, published in peer-reviewed journals between 1/1/1975 and 1/6/2015.
(ii)  RCTs with a comparison arm or control group that constituted usual care.
(iii)   Human participants older than 18 years of age with clinically confirmed type 2 diabetes, at time of recruitment.
(iv)   Primary clinical outcome measure was HbA_1c_, however studies reporting HbA_1c_ results as an outcome measure were also included. Body weight was reported as a secondary outcome (because of the inconsistency and variety of measures of dietary and physical activity behaviour used in the RCTs, it was not possible to compare behavioural outcomes across trials. Thus, HbA_1c_ was selected as the primary endpoint).

### Exclusion criteria

(i) RCTs of diabetes prevention OR RCTs of those at risk of type 2 diabetes.
(ii)  RCTs that used pharmacological agents exclusively to treat type 2 diabetes.
(iii)   RCTs that targeted multiple chronic diseases, gestational diabetes or type 1 diabetes.
(iv)   RCTs that used additional interventions beyond diet and physical activity, or focused on additional behaviours other than diet and physical activity.
(v)  Studies not reported in English.
(vi)   Studies not reporting HbA_1c_ as an outcome measure.

### Information sources and search strategy

Cochrane Library, CINAHL, EMBASE, PubMed, PsycINFO, and SCOPUS databases were systematically searched using a Boolean combination of key words and MeSH headings (Additional file 1: 1.2). Additional records identified through other sources such as reference lists of relevant reviews and included studies were searched for additional studies. The original search was conducted in April 2014 and repeated June 2015. Reference lists of included articles were also checked for relevant articles.

### Article screening

**Fig. 1:**
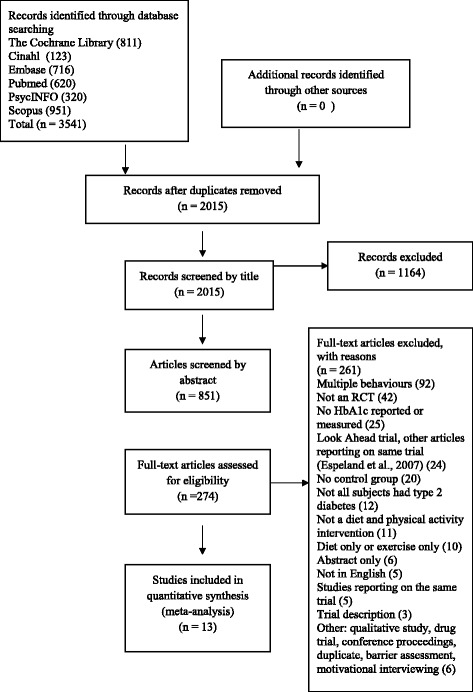
PRISMA 2009 Flow diagram of search strategy

Articles were initially screened by two research team members based on titles and abstracts and then full texts of the remaining articles (KC and KMG). The final set of included articles was agreed on by the entire team (see Fig. 1 for search process). Inter-rater agreement by Cohen’s Kappa for the full text search results was 0.86.

### Data extraction process

Data were extracted using standardised data extraction templates and compiled in an Excel file. All data extraction was carried out independently by at least two members of the team (KC and KMG). If additional study information was required, corresponding authors were contacted by email using a standardised template, papers reporting on the same trial were sought (e.g. Methods papers), and when available, supplementary online information was accessed.

### Risk of bias and fidelity assessment

Risk of bias in individual studies was assessed using the Cochrane Collaboration risk of bias tool, [23] whereby criteria are applied to seven aspects of trials to yield an appraisal of ‘low risk’, ‘high risk’ or ‘unclear risk’ of bias. RCTs were independently assessed by two members of the review team for methodological quality and risk of bias (KC and KMG). Treatment fidelity was assessed using Bellg et al.’s [24] criteria, which identify treatment fidelity strategies for improving and monitoring, provider training, delivery of treatment, receipt of treatment, and enactment of treatment skills. Each category contains subcategories which were each assigned a score of yes, no, or unclear. However, fidelity measures using this dichotomous type response don’t capture the degree of use of fidelity, therefore a continuum type scoring or rating of parameters may provide a more accurate assessment of fidelity.

### Coding of behaviour change techniques

Michie’s v1 BCT taxonomy [25] was used to identify and code the BCTs reported in each study. This rigorously developed and validated taxonomy consists of clear definitions of 93 different BCTs, divided into 16 different categories. The taxonomy was developed to facilitate consistent classification and reporting of the use of BCTs by researchers and clinicians. Since its publication, it has become the standard for classifying and reporting BCTs in the health behaviour change literature. BCTs were coded separately for physical activity behaviour and for diet behaviour; a BCT was only coded when it was explicitly mentioned in the intervention methodology. (All studies coded and associated text are documented in Additional file 2). BCTs were coded separately for intervention and control groups. BCTs for diet only and physical activity only were combined in an excel spreadsheet, if a BCT was present in diet only or physical activity only or in both diet and physical activity it is reported as present for combined diet and physical activity (see Table 1). A coding rubric/rulebook was developed by three authors of this review (KC, LQ and HG) to guide the coding process (Additional file 1: 1.3). All included studies were coded independently by two authors (KC and LQ) who underwent training in the use of Michie’s taxonomy [26]. A third master coder (HG) independently assessed the coding results and had final say in the event of disagreements. Cohen’s kappa and PABAK calculations were used to establish inter-coder reliability of BCTs present and absent. A BCT had to be used in at least three studies to be included in the moderator analysis.

**Table 1:** BCTs used in dietary AND physical activity aspect of intervention

BCT no.	BCT Label	(1)	(2)	(3)	(4)	(5)	(6)	(7)	(8)	(9)	(10)	(11)	(12)	(13)	Total
4.1	Instruction on how to perform a behaviour	✓	✓	✓	✓	✓	✓	✓	✓	✓	✓	✓	✓	✓	13
1.4	Action planning	✓	✓	✓	✓	✓	✓	✓	✓	✓	✓	✓	✓		12
9.1	Credible source	✓	✓	✓	✓	✓	✓	✓	✓		✓	✓	✓	✓	12
1.1	Goal setting (behaviour)	✓	✓	✓	✓	✓	✓		✓	✓	✓	✓	✓		11
1.3	Goal setting (outcome)	✓	✓	✓	✓		✓	✓			✓	✓	✓	✓	10
3.1	Social support (unspecified)	✓	✓	✓	✓	✓				✓	✓	✓	✓	✓	10
2.3	Self-monitoring of behaviour	✓	✓	✓	✓	✓	✓	✓	✓		✓				9
2.2	Feedback on behaviour	✓		✓	✓			✓	✓	✓		✓			7
6.1	Demonstration of the behaviour	✓		✓	✓	✓		✓		✓		✓			7
8.7	Graded tasks	✓	✓	✓	✓	✓	✓	✓							7
12.5	Adding objects to the environment		✓	✓	✓	✓		✓	✓	✓					7
1.2	Problem solving	✓		✓	✓	✓					✓				5
2.5	Monitoring outcome(s) of behaviour by others without feedback	✓	✓		✓				✓					✓	5
8.1	Behavioural practice/rehearsal	✓			✓	✓		✓				✓			5
12.3	Avoidance/reducing exposure to cues for the behaviour	✓		✓	✓	✓									4
1.5	Review behaviour goal(s)				✓			✓				✓			3
1.7	Review outcome goal(s)		✓		✓					✓					3
2.4	Self-monitoring of outcome(s) of behaviour		✓	✓	✓										3
2.7	Feedback on outcome(s) of behaviour			✓	✓					✓					3
12.1	Restructuring the physical environment			✓	✓	✓									3
2.1	Monitoring of behaviour by others without feedback								✓				✓		2
3.3	Social support (emotional)			✓		✓									2
5.1	Information about health consequences				✓	✓									2
6.2	Social comparison				✓	✓									2
7.1	Prompts/cues			✓	✓										2
8.2	Behaviour substitution				✓						✓				2
8.6	Generalization of a target behaviour			✓								✓			2
10.3	Non-specific reward			✓	✓										2
10.9	Self-reward			✓	✓										2
15.4	Self-talk			✓	✓										2
1.6	Discrepancy between current behaviour and goal			✓											1
2.6	Biofeedback				✓										1
3.2	Social support (practical)				✓										1
7.5	Remove aversive stimulus				✓										1
8.3	Habit formation				✓										1
9.2	Pros and cons				✓										1
10.2	Material reward (behaviour)				✓										1
10.4	Social reward				✓										1
10.6	Non-specific incentive				✓										1
10.7	Self-incentive				✓										1
11.2	Reduce negative emotions				✓										1
12.2	Restructuring the social environment				✓										1
13.1	Identification of self as role model				✓										1
13.2	Framing/reframing				✓										1
15.1	Verbal persuasion about capability				✓										1
15.3	Focus on past success				✓										1

Studies are listed in alphabetical order. (1) [43], (2) [45], (3) [46], (4) [38], (5) [37], (6) [41], (7) [72], (8) [44], (9) [40], (10) ([39], (11) [42], (12) [36], (13) [47]

### Coding of intervention features

Rationale for features included was derived from intervention features identified previously [27], previous reviews [7, 17] and the ‘Theory Coding Scheme’ [28] which guided theory coding of intervention content. Intervention features were included under the headings “mode of delivery”, “frequency”, “provider”, “intensity” and “other” (use of theory and baseline HbA_1c_, number of BCTs included). Intensity for total number of contacts and total number of face-to-face contacts with intervention personnel used the mean and median to categorise variables into high (above mean/median) and low intensity (below mean/median). Frequency of ‘total’ and ‘face -to-face’ contacts also used above and below the mean/median to categorise the average number of weeks between contacts as high frequency (below) and low frequency (above). All other intervention features were analysed dichotomously using yes/no to indicate presence or absence. Rationale for categorising baseline HbA_1c_ levels comes from a large epidemiology study which identified that HbA_1c_ levels ≥ 7 % were associated with increased risk of death [29]. We also ran the moderator analysis using above and below 8 % (64 mmol/mol) to categorise high and low HbA_1c_ as standard diabetes control targets aim to keep HbA_1c_ between 7.0 and 7.9 % [29] therefore HbA_1c_ levels >8 % represent poorly controlled type 2 diabetes.

### Analysis

HbA_1c_ reductions of ≥0.3 % were deemed clinically significant, which follows the precedent set by other authors [6, 7]. Meta-analyses were conducted using RevMan (v5.3) on the primary outcome measure of HbA_1c_ and the secondary outcome of body weight. Changes were calculated as the difference in HbA_1c_ from baseline to a particular time-point (3, 6, 12, and 24 months), and reductions in HbA_1c_ were calculated as the difference between intervention and control groups. Means and standard deviations (SDs) from included studies were converted to mean differences and SDs of the differences between intervention and control groups at 3, 6, 12 and 24 months.

### Meta-analysis

Missing SDs were calculated from SE, t and *p* values, using the Cochrane guidelines [30]. The mean for one study was estimated from the median and range using Hozo’s formula [31]. The SD of the difference in means from baseline to the different time points was calculated using the Cochrane guidelines when standard error or 95 % confidence intervals were reported. A strategy documented by previous researchers, which requires a correlation between baseline and end of intervention measurements, was used for the remaining missing data [32, 33]. A correlation of 0.75 was used to calculate the missing SDs for HbA_1c_ data; this value was chosen following a sensitivity analysis using correlations of 0.5, 0.75 and 0.95, and a previous review and meta-analysis [34]. A correlation of 0.95 was used to calculate the missing SDs for weight loss data, following a further sensitivity analysis and previous studies [33, 35]. We also calculated the SDs of the difference between baseline and reported time-point means for three studies that reported sufficient data to calculate, and this was consistent with the correlations we used. As this correlation is only an estimate as the raw data was unavailable, it is also suggested that future researchers use the Bayesian principle of combining raw data from similar previously published studies to, calculate missing SDs where available and combine these results on similar subjects to improve the accuracy of this estimation. It was estimated that the HbA_1c_ and weight loss variance is the same at baseline and reported time points for the control and the intervention groups when variance was not reported. Effect heterogeneity was assessed using the I^2^ method using the Cochrane guidelines [30]. For the overall meta-analysis, data reported at the time point closest to the end of the intervention was used (cf., Avery et al. [7]). A random effects analysis model using the inverse variance statistical method was used. A repeated measures design was not possible as the raw data were unavailable. Statistical significance of the moderator and meta-analysis was set at *p* ≤ 0.05.

### Moderator analysis

A moderator analysis was conducted to identify associations between BCTs, intervention features and changes in HbA_1c_ using Comprehensive Meta-Analysis (V3). All studies were combined using data reported at the time point closest to the end of the intervention. The BCTs used for both diet and physical activity aspects of interventions were combined for one meta-analysis where BCTs were included if present in diet only or physical activity only or in both. The moderator analysis used the effect size ‘difference in means’ to assess the data, and carried out subgroup analysis of the included studies, comparing presence or absence of BCTs or intervention features. A separate moderator analyses were also conducted for dietary BCTs and for physical activity BCTs. BCTs present in the control group were not included in the moderator analysis. A random effects model was used to analyse the data.

## Results

### Study selection and study characteristics

Thirteen studies met the inclusion/exclusion criteria. Summary characteristics of included studies are outlined in Additional file 1: 1.4. One study [36] reported data for males and females separately so these data are presented as a mean of both groups. Average age of participants was 56.7 (±3.9) years for intervention groups and 56.8 (±3.9) years for controls. For intervention and control groups respectively, mean duration of diabetes, where reported, was 6.9 (± 1.2) and 8 years (± 3), mean baseline HbA_1c_ 8.03 % (± 1.21 %) and 8 % (± 0.95 %), weight 88.5 kg (± 14.5 kg) and 87.9 kg (± 14.8 kg). Only one of the included studies [37] was carried out in a community centre setting, all remaining studies were carried out in a clinical setting. All participants included in the thirteen studies were classified as having type 2 diabetes.

### Risk of bias and treatment fidelity

Only one RCT was judged as low risk of bias in each of the seven areas assessed [38]. Nine RCTs were judged to have a combination of low and unclear risk of bias apart from three RCTs which were judged to have a high risk of bias in the ‘other bias’ category, [37, 39] ‘blinding of participants and personnel’ and ‘blinding of outcome assessment’ categories [40] (Additional file 1: 1.5, 1.6). Inter-rater agreement (0.86) was determined by Cohen’s kappa for risk of bias assessment. Results of the assessment of treatment fidelity are presented in Additional file 1: 1.7. Overall reported use of treatment fidelity strategies was very low across all categories apart from ‘monitoring and improving enactment of treatment skills’ where 11 out of 13 studies scored ‘yes’ in the subcategory ‘ensuring participants’ use of behavioural skills’. Coding of all subcategories is more comprehensive, however, fidelity assessment is much lower using this method.

### Meta-analysis of changes in HbA_1c_ and body weight

**Fig. 2:**
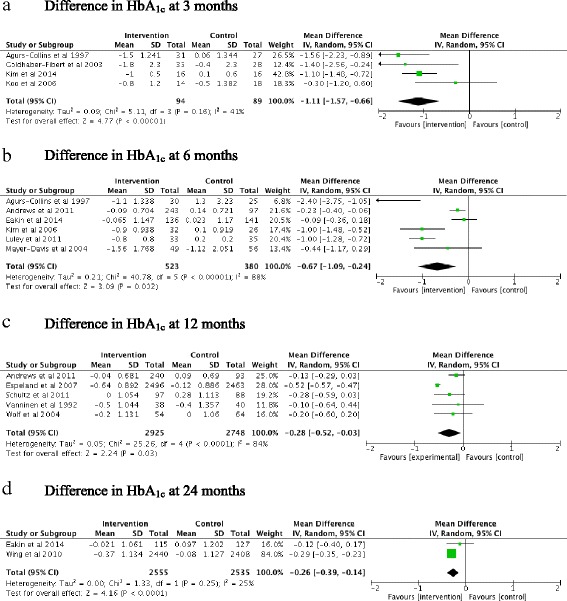
Meta analyses of HbA_1c_ changes (%) at 3 (**a**), 6 (**b**), 12 (**c**) and 24 (**d**) months

**Fig. 3:**
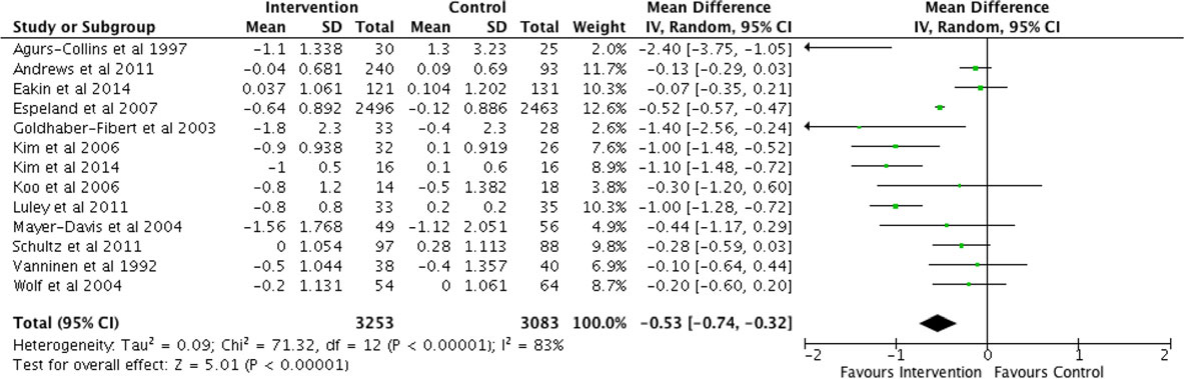
Overall meta-analysis of mean difference in HbA_1c_ (%) from baseline. (studies with multiple time points are represented by time point closest to the end of intervention)

Meta-analyses showed differences in HbA_1c_ between intervention and control groups of -1.11 % (12 mmol/mol [95 % CI -1.57 to -0.66, *P* < 0.00001]), -0.67 % (7 mmol/mol [95 % CI -1.09 to -0.24 *P* = 0.002]), -0.28 % (3 mmol/mol [95 % CI -0.52 to -0.03, *P* = 0.03]), and -0.26 % (2 mmol/mol [95 % CI -0.39 to -0.14, *P* < 0.001]), at 3 (*n* = 4), 6 (*n* = 6), 12 (*n* = 5) and 24 (*n* = 2) months respectively (Fig. 2). When all studies and all time points were included in an overall meta-analysis, reduction in HbA_1c_ was 0.53 % (6 mmol/mol [95 % CI -0.74 to -0.32, *P* < 0.00001]) (Fig. 3). Sensitivity analysis showed the magnitude of reduction did not change whether data from time point closest to end of intervention or final time point reported was used in analysis. Heterogeneity as measured by I^2^ was 41 %, 88 %, 84 % and 25 % at 3, 6, 12 and 24 months respectively.

The difference in body weight between intervention and control groups was -2.7 kg (-4.14 -1.26, *P* = 0.06), -3.64 kg (-6.05 to -1.23, *P* = 0.003), -3.77 kg (-7.77 to 0.22, *P* = 0.06), and -3.18 kg (-7.67 to 1.32, *P* = 0.17), at 3, 6, 12 and 24 months respectively (Additional file 1: 1.8). Overall meta-analysis for body mass showed a reduction of -3.73 kg (-6.09 to -1.37, *P* = 0.002), (Additional file 1: 1.9). Heterogeneity as measured by I^2^ was 60 %, 91 %, 97 % and 98 % at 3, 6, 12 and 24 months respectively.

### Diet and physical activity content of interventions

The majority of included studies focused on a reduction of calories (10 of 13), three studies did not specify the caloric goal of their intervention [37, 41, 42]. There was an additional focus on low fat [39, 43], low carbohydrate [40, 44] and low glycaemic index [45] in some of the included studies. All of the included studies (*n* = 13) focused on aerobic exercise of a moderate intensity, three also focused on strength training [38, 42, 46] (Additional file 1: 1.10).

### BCTs used

Inter-rater agreement determined by Cohen’s kappa was 0.79 and PABAK was 0.92 (Additional file 1: 1.11). A total of 46 different BCTs were applied in the intervention groups. Sixteen of these 46 BCTs were reported only once. The number of BCTs used in a single RCT ranged from 5 [47] to 42 [38], with a mean of 13.5 (median 11). Individual BCTs and their frequency of use are reported for combined diet and/or physical activity behaviour in Table 1. Control group BCTs were coded separately, four different BCTs were identified with ‘instruction on how to perform a behaviour’ (*n* = 6) the most frequently occurring. BCTs coded for diet only and physical activity only are reported in Additional files 1: 1.12 and 1.13. BCT analysis by category and BCTs not used are presented in Additional files 1: 1.14 and 1.15. BCTs coded and text rationale for all studies is documented in Additional file 2.

### Moderator analysis of BCTs

Moderator analysis showed four BCTs for both behaviours associated with > 0.3 % reduction in HbA_1c_. Presence of the BCTs ‘instruction on how to perform a behaviour’ (-0.549 %), ‘behavioural practice/rehearsal’ (-0.417 %), ‘action planning’ (-0.385 %) and ‘demonstration of the behaviour’ (-0.343), were associated with clinically significant reductions in HbA_1c_. Seven other BCTs were associated with reductions in HbA_1c_ with the BCTs ‘graded tasks’ (-0.217 %), and ‘feedback on behaviour’ (-0.203 %) showing the strongest association but these were not clinically or statistically significant (Table 2).

**Table 2:** Moderator analysis of HbA_1c_ for diet AND physical activity BCTs

			Effect size 95 % CI			Effect size 95 % CI			Subgroup analysis		
BCT No.	BCTs	k present (absent)	Present	Lower limit	Upper limit	Absent	Lower limit	Upper limit	Q	P	Difference
4.1	Instruction on how to perform a behaviour	13 (0)	−0.549	−0.762	−0.337				0	1	−0.549
8.1	Behavioural practice/rehearsal	5 (8)	−0.833	−1.251	−0.415	−0.416	−0.733	−0.1	2.423	0.12	−0.417
1.4	Action planning	12 (1)	−0.585	−0.811	−0.36	−0.2	−0.922	0.522	0.996	0.318	−0.385
6.1	Demonstration of the behaviour	7 (6)	−0.701	−0.997	−0.405	−0.358	−0.702	−0.013	2.201	0.138	−0.343
8.7	Graded tasks	7 (6)	−0.653	−0.96	−0.346	−0.436	−0.785	−0.087	0.833	0.361	−0.217
2.2	Feedback on behaviour	7 (6)	−0.641	−0.939	−0.343	−0.438	−0.792	−0.084	0.74	0.39	−0.203
12.3	Avoidance/reducing exposure to cues for the behaviour	4 (9)	−0.694	−1.209	−0.179	−0.53	−0.848	−0.212	0.283	0.595	−0.164
2.3	Self-monitoring of behaviour	9 (4)	−0.612	−0.894	−0.329	−0.453	−0.846	−0.06	0.414	0.52	−0.159
1.2	Problem solving	5 (8)	−0.647	−1.111	−0.183	−0.539	−0.869	−0.208	0.139	0.709	−0.108
1.5	Review behaviour goal(s)	3 (10)	−0.618	−1.09	−0.145	−0.551	−0.859	−0.242	0.054	0.816	−0.067
12.5	Adding objects to the environment	7 (6)	−0.565	−0.854	−0.276	−0.542	−0.9	−0.183	0.01	0.921	−0.023
1.7	Review outcome goal(s)	3 (10)	−0.536	−0.943	−0.129	−0.573	−0.861	−0.284	0.021	0.884	0.037
2.7	Feedback on outcome(s) of behaviour	3 (10)	−0.53	−0.977	−0.082	−0.585	−0.888	−0.282	0.04	0.841	0.055
1.1	Goal setting (behaviour)	11 (2)	−0.53	−0.772	−0.289	−0.654	−1.17	−0.138	0.182	0.67	0.124
12.1	Restructuring the physical environment	3 (10)	−0.47	−1.022	0.081	−0.61	−0.923	−0.297	0.186	0.666	0.14
2.5	Monitoring outcome(s) of behaviour by others without feedback	5 (8)	−0.44	−0.818	−0.061	−0.639	−0.942	−0.336	0.647	0.421	0.199
1.3	Goal setting (outcome)	10 (3)	−0.472	−0.697	−0.247	−0.908	−1.408	−0.409	2.437	0.118	0.436
2.4	Self-monitoring of outcome(s) of behaviour	3 (10)	−0.251	−0.633	0.131	−0.714	−0.99	−0.438	3.71	0.054	0.463
3.1	Social support (unspecified)	10 (3)	−0.45	−0.678	−0.221	−0.92	−1.372	−0.468	3.309	0.069	0.47
9.1	Credible source	12 (1)	−0.491	−0.709	−0.274	−1	−1.627	−0.373	2.254	0.133	0.509

Meta-analysis (random effects model was used to assess the data)

When the moderator analysis was run separately for dietary BCTs, the BCT ‘demonstration of the behaviour’ was associated with clinical and statistically significant reductions in HbA_1c_. The BCTs ‘behavioural practice/rehearsal’ and ‘instruction on how to perform a behaviour’, were associated with clinically significant reductions (Additional file 1: 1.16). Moderator analysis for physical activity showed three BCTs associated with clinically significant reductions in HbA_1c_, ‘instruction on how to perform a behaviour’, ‘credible source’ and ‘behavioural practice/rehearsal’ (Additional file 1: 1.17). Moderator analysis of intervention features are documented in Table 3.

**Table 3:** Moderator analysis of intervention features for diet and physical activity

		Effect size 95 % CI			Effect size 95 % CI			Subgroup analysis		
Intervention Features	k present (absent)	Present	Lower limit	Upper limit	Absent	Lower limit	Upper limit	Q	P	Difference
Mode										
Supervised physical activity component	5 (8)	−0.94	−1.323	−0.558	−0.368	−0.631	−0.106	5.852	0.016	−0.572
Individual face to face	6 (7)	−0.545	−0.885	−0.204	−0.576	−0.905	−0.247	0.017	0.897	0.031
Group sessions only	5 (8)	−0.856	−1.218	−0.495	−0.408	−0.643	−0.172	4.16	0.041	−0.448
Combination of group and individual sessions	4 (9)	−0.545	−1.013	−0.077	−0.588	−0.914	−0.263	0.022	0.881	0.043
Individual contact only	4 (9)	−0.349	−0.712	0.015	−0.661	−0.93	−0.393	1.841	0.175	0.312
Frequency										
Frequency of total contacts (median = 1.73)^a^	7 (6)	−0.828	−1.083	−0.574	−0.17	−0.456	0.116	11.358	0.001	−0.658
Frequency of total contacts (mean 2.61)^a^	10 (3)	−0.705	−0.932	−0.479	−0.101	−0.469	0.268	7.501	0.006	−0.604
Frequency of face to face contacts (median 1.96)^a^	6 (7)	−0.934	−1.316	−0.552	−0.313	−0.627	0.001	6.061	0.014	−0.621
Frequency of face to face contacts (mean 3.13)^a^	8 (5)	−0.764	−1.089	−0.438	−0.322	−0.678	0.034	3.224	0.073	−0.442
Provider										
Contact with exercise physiologist, trainer	6 (7)	−0.762	−1.124	−0.401	−0.398	−0.73	−0.066	2.12	0.145	−0.364
Combination of dietitian and exercise physiologist	4 (9)	−0.778	−1.222	−0.334	−0.466	−0.778	−0.155	1.272	0.259	−0.312
Contact with dietitian/ nutritionist	10 (3)	−0.488	−0.677	−0.219	−0.886	−1.316	−0.455	3.093	0.079	0.398
Interventionist other than dietitian, exercise physiologist, i.e. nurse, doctor	4 (9)	−0.477	−0.848	−0.046	−0.628	−0.928	−0.327	0.5	0.48	0.151
Intensity										
Intensity: number of face to face contacts (median (16)^a^	7 (6)	−0.804	−1.144	−0.465	−0.32	−0.66	0.02	3.9	0.048	−0.484
Intensity: number of face to face contacts (mean (20.2)^a^	4 (9)	−0.784	−1.261	−0.307	−0.481	−0.79	−0.172	1.092	0.296	−0.303
Intensity: number of total contacts with intervention personnel (median (25.5)^a^	7 (6)	−0.609	−0.905	−0.314	−0.479	−0.842	−0.117	0.297	0.585	−0.13
Intensity: number of total contacts with intervention personnel (mean (29.2)	5 (8)	−0.75	−1.075	−0.426	−0.39	−0.684	−0.097	2.599	0.107	−0.36
Other										
Use of theory/model to inform intervention	3 (10)	−0.483	−0.994	0.029	−0.567	−0.807	−0.327	0.086	0.769	0.084
Baseline HbA1c levels >8%^b^	5 (8)	−0.943	−1.397	−0.49	−0.441	−0.677	−0.205	3.707	0.054	−0.502
Baseline HbA1c levels >7%^b^	12 (1)	−0.608	−0.837	−0.379	−0.13	−0.754	0.494	1.983	0.159	−0.478
Number of BCT's Median (11)^c^	6 (7)	−0.469	−0.806	−0.131	−0.627	−0.932	−0.323	0.469	0.494	0.158
Number of BCT's Mean (14.85)^c^	4 (9)	−0.694	−1.209	−0.179	−0.53	−0.848	−0.212	0.283	0.595	−0.164

Meta-analysis (random effects model was used to assess the data)

^a^Present denotes higher frequency/intensity, absent denotes lower frequency/intensity, above and below mean/median

^b^Present denotes high baseline HbA1c, absent denotes lower HbA1c, above and below mean/median

^c^Present denotes higher number of BCTs, absent denotes lower number of BCTs, above and below mean/median

## Discussion

We found significant mean reductions in HbA_1c_ at three and six months but not at 12 or 24 months. Reductions in body weight were observed at all time points and were greatest at 12 months. Results revealed four BCTs and nine intervention features associated with clinically significant reductions in HbA_1c_ (> 0.3 %). These findings are exploratory but lay a foundation for future hypotheses with clinical and research implications.

### Combining diet and physical activity

Overall HbA_1c_ results of this review highlight the value of combining diet and physical activity and the difficulty in maintaining initial reductions in HbA_1c_ over time. Diet and physical activity interventions produced superior results in our review (-0.53 %) and other reviews (-0.58 %) [48] compared to physical activity only, [7] dietary treatment only, [49] computer based interventions [50] and psychological interventions [51]. Reviews have shown that physical activity was associated with a reduction in HbA_1c_, but only when combined with diet [48, 52]. Our observed reduction in weight (3.73 kg) is similar to other reviews of 3.2 kg [53], 3.0 kg [13] and 3 to 5 kg [52] in those at risk of type 2 diabetes but greater than reviews of diet only: low-carbohydrate (0.69 kg) or Mediterranean diets (1.84 kg) [49]. A meta-analysis reported that a physical activity and behavioural intervention in addition to a diet intervention lost 3 kg more weight than diet only and even greater weight losses were achieved with higher intensity physical activity [34].

Most interventions in type 2 diabetes focus on multiple rather than single behaviour change [54], however changing multiple behaviours simultaneously is difficult [55]. Changing multiple behaviours simultaneously rather than changing behaviours individually has been found to be more effective in changing at least one behaviour [55]. The mechanistic basis for this is unclear. The extent to which diet and physical activity interventions interact synergistically is also unclear. It has been suggested that successful behaviour change in one behaviour can facilitate change in other behaviours and it may be more appropriate to target behavioural patterns [56]. A qualitative study suggested that physical activity plays a greater supporting role for dietary behaviour change than dietary behaviour change did for physical activity, and should be the first behaviour individuals are encouraged to change [57], however, a study comparing sequential versus simultaneous delivery concluded that simultaneous delivery of diet and physical activity programmes may yield the most effective outcomes [58].

## BCTs

### Frequently used and number of BCTs

The most frequently used BCTs in diet and physical activity interventions may not be the most effective. Eleven BCTs showed a reduction in HbA_1c_, however only six of these were among the ten most frequently used BCTs suggesting that only 60 % of the most frequently used BCTs were effective which could have important implications for intervention study design, resource utilisation and cost effectiveness. A review of physical activity interventions showed that only 50 % of the most frequently used BCTs were associated with reductions in HbA_1c_ [7]. It’s possible that less frequently reported BCTs not included in the moderator analysis (*n* = 26) are associated with reductions in HbA_1c_. Another possible conclusion could be that certain BCTs are necessary but not sufficient elements of interventions and perhaps the presence of certain BCTs is required for the key BCTs to work as intended. Our work suggests that researchers need to conduct a detailed behavioural diagnosis prior to designing their interventions, possibly using a framework such as Michie et al.’s COM-B, to align BCTs with sources of behaviour, intervention functions and policy categories as different BCTs may be more appropriate for certain individuals, behaviours, personalities, psychological profiles or different modes of delivery.

Improvements in HbA_1c_ were also associated with the use of a greater number of BCTs in this review also observed in other studies using HbA_1c_ [7] and weight loss as outcomes [12, 13]. However, how using a greater or lesser number of BCTs in intervention studies can affect outcomes remains unclear and requires further investigation [13]. The number of BCTs used is inextricably linked to quality of reporting and the fidelity of use of BCTs. Greater treatment fidelity and quality reporting of interventions will enhance confidence, robustness and study power of reported results [59].

### BCTs associated with reductions in HbA_1c_

We identified four BCTs associated with clinically significant reductions in HbA_1c_: ‘instruction on how to perform a behaviour’, ‘behavioural practice/rehearsal’ ‘action planning’ and ‘demonstration of the behaviour’. These have all been reported previously as having a positive impact on diet and physical activity behaviour [13, 14, 17]. Usually the three BCTs: ‘instruction on how to perform a behaviour’, ‘behavioural practice/rehearsal’ and ‘demonstration of the behaviour’ are coded together when delivered through classes such as exercise or cookery. This coding principle might explain the emergence of these three BCTs as key to changing diet and physical activity behaviour as it’s possible that these three BCTs work in isolation but more likely that the presence of all three allows them to work synergistically. This also highlights that some BCTs lend themselves well to certain modes of delivery. Success of these three BCTs might be explained by their strong theoretical foundations [60, 61]. The Social Cognitive Theory includes ‘observational learning’ as one of its five basic capabilities of human functioning [61]. The ‘vicarious capability’ suggested in this model outlines our ability to learn through observation and modeling behaviour of others and is intertwined in these three BCTs and a review of nutrition counseling strategies suggested including skill development coaching/training and demonstration or modeling [18].

One BCT from the ‘goals and planning’ category, ‘action planning’ was associated with clinically significant reductions in HbA_1c_. This BCT has also been associated with successful behaviour change in several other studies [7, 12–15, 18]. The BCT ‘action planning’ facilitates behaviour change by providing a clear pathway in identifying context, frequency, duration and intensity of the required behaviour change. Constructs from this BCT highlight the importance of self-regulatory processes in behaviour change [62] and can be seen in several behaviour change theories [63, 64].

Two BCTs from the ‘feedback and monitoring’ category ‘feedback on behaviour’ and ‘self-monitoring of behaviour’ were associated with reductions in HbA_1c_. These BCTs have also been associated with successful behaviour change in other studies [12–16, 18] and similar constructs are described in a theoretical model [61]. BCTs in this category can help keep the behaviour change on track, allow for adjustment and self-regulation and may be more important in maintaining than initiating behaviour change as it’s necessary to self-monitor behaviour to self-regulate behaviour [62]. As motivation decreases and opportunity costs increase, there is a greater need for self-regulatory effort [65]. However, according to the Control Theory [66] the self-regulation process of how we set and prioritize our goals is based on a hierarchical structure. It’s also thought that the self-regulatory process or willpower to sustain behavioural change draws on a mental resource requiring energy and one which can be depleted, making subsequent tasks more difficult [67].

Several authors have highlighted the benefits of using the BCTs ‘goal setting’ [7, 12, 18] ‘goal review’, ‘social support’ [12], ‘prompt practice’ [13], ‘use of follow up prompts’ [15, 18] and ‘prompting generalisation of a target behaviour’ [7] to positively affect behaviour change of diet and/or physical activity. However, these findings were not observed in our review, possibly due to limitations outlined in this study or limitations in reporting.

### BCTs not used and other factors

Some of the best established BCTs [25, 26] for behaviour change were conspicuous in their absence from any of the RCTs in this review. These included ‘behavioural contract’ and ‘commitment’. Behaviour change is almost impossible without a high level of commitment. Interventions could benefit from assessing levels of commitment prior to intervention. Lesser-used categories ‘Reward and threat’ and ‘Identity’ could also represent opportunities for behaviour change [14, 68] as identity represents one of the strongest drivers for behaviour change, and has been associated with positive changes in health outcomes, [68, 69] as did BCTs using automatic process such as ‘habit formation’ and ‘habit reversal’ [70].

It is also possible that some BCTs have a negative effect on behaviour. In this review presence of four BCTs ‘goal setting (outcome)’, ‘self-monitoring of outcomes of behaviour’, ‘social support’ and ‘credible source’, were associated with clinically significant increases in HbA_1c_. Although the ‘credible source’ BCT data are heavily skewed by one study, evidence suggests that monitoring outcomes of behaviour and setting outcome-related goals may negatively affect diet and/or physical activity behaviour. This finding warrants further investigation.

Another factor not considered in this review is the study of epigenetics, the complex relationship between the environment and genes [71] and to what extent diet and physical activity behaviours may be genetically determined and influenced.

### Intervention features

This review identified nine intervention features associated with clinically significant reductions in HbA_1c_. Interventions where the physical activity component was supervised (*n* = 5) showed one of the strongest moderating effects with both aerobic [37, 41, 43] and strength based activities [42, 72]. Interventions that use ‘group sessions only’ were associated with greater effectiveness than those with individual sessions only. However, higher frequency and intensity of individual contact was associated with greater effectiveness. Evidence suggests that females may benefit more from group sessions [73] while males may benefit more from individual sessions [74].

Our findings suggest that diet and physical activity interventions delivered by an exercise physiologist or an exercise physiologist and a dietitian through face-to-face contact may be the best way to deliver these interventions, though cost-effectiveness was not assessed. Interventions delivered by non-diet or exercise specialists (doctor, nurse) were not associated with success, which suggests that diet and/or physical activity interventions need to be delivered by experts in that area. While app delivered interventions hold promise, [75] our findings suggest that frequent personal contact and supervised physical activity may enhance effectiveness.

A gradual increase in intensity and frequency of contact could well assist in achieving maintenance of behaviour change of diet and physical activity as simple tasks in the initial stages of interventions, gradually progressing in intensity, could help improve participants’ self-efficacy [76, 77]. Three out of four interventions reporting multiple time points reported that initial reductions in HbA_1c_ were not maintained [38, 43, 46]. The increased effectiveness of gradually increasing interventions may also be explained by their role in tackling habituation, or boredom, or providing increased support as behaviour change becomes more challenging following the initial stages.

Our review suggests that the BCT ‘graded tasks’ was associated with a reduction in HbA_1c_, and positive health outcomes in another review [78]. The BCT ‘graded tasks’ can play a key role in developing habits which is among the five theoretical themes suggested for behavioral change maintenance [65] and may inform better maintenance of behaviour change in diet and physical activity interventions.

### Use of theory

Only three out of 13 RCTs mentioned use of a theory or model in designing intervention [39, 43, 46]. It wasn’t possible to ascertain to what degree these studies were guided by theory as fidelity to theory was not reported. One study [43] reported that the behavioural component ‘was based on’ the Social Action theory [79], a second study [39] reported that they used ‘concepts’ from this theory, while another [46] reported that methods used were ‘grounded’ in the Social Cognitive Theory [61]. In evaluating and developing complex interventions, a strong theoretical understanding is required to identify and strengthen the weakest links in the causal chain [80]. Interventions guided by theory or theoretical constructs may be more effective in changing a variety of health behaviours than studies not using theory [81]. However, a study of the extent and use of theory in physical activity and healthy eating interventions suggested that theories were not used extensively in the development of interventions and when theory was used the relationship between effectiveness and extent and use of theory was weak [82] which is corroborated by data from this review.

### Study strengths and limitations

We used the most recent BCT taxonomy (v1) to code interventions. To maximise the quality of the research being reviewed only RCTs were included. The detailed reporting of outcomes of HbA_1c_ and reduction in body weight at different time points allow for investigation of effect size and trends over time. The systematic detailing of BCT coding procedures, results, and high inter-rater reliability allows future researchers to replicate and review methods used in detail. The overall risk of bias was low. This review is, to the best of our knowledge, the first to document key BCTs and intervention features associated with reductions in HbA_1c_ in diet *and* physical activity interventions for type 2 diabetes.

Some limitations also warrant mention. Results of this review can be considered exploratory as no causality of BCTs/intervention features associated with clinically significant reductions in HbA_1c_ can be concluded, and the presence of a BCT can only infer association. The strict inclusion criteria limited the review to 13 studies, and large heterogeneity reduced study power and robustness of results in elucidating HbA_1c_ effect sizes. Coding of the BCTs depended on the reporting quality, quantity, and accuracy within the RCTs themselves, and these varied considerably. For instance, regarding the Look Ahead Trial [38, 83], when the RCT results paper was coded, 11 BCTs were identified; when the methodology paper was coded, 16 BCTs were identified [84]; however, when all 88 supporting documents (https://www.lookaheadtrial.org/) were coded, 42 BCTs were identified. A study of smoking interventions showed similar results [85]. The majority of reviewed studies did not reference an associated methodology paper, rendering it possible that other BCTs were used but not coded. Fidelity was poorly reported, therefore, it was not possible to determine if BCTs were delivered, received or enacted as intended. It was not possible to code the dose, frequency or sequence of use of BCTs or to ascertain which BCTs were associated with initiation or maintenance of behaviour change. Comparisons drawn between this review and previous studies should take into account the different BCT Taxonomies used [25, 86–88]. Variation between studies in subject’s duration of diabetes and baseline HbA_1c_ may also have increased heterogeneity. The majority of the included studies did not report behaviour change for diet or physical activity as an outcome measure.

### Implications and future directions

From a research perspective we recommend that a formal assessment of the effectiveness of individual and clustered BCTs in the initiation and maintenance of behaviour change should be a scientific priority. The hierarchical ranking of BCTs and the synergistic effect of certain BCTs requires further investigation. We recommend firstly that clearly defined and reported behavioural outcome measures are incorporated into diet and or physical activity interventions and studies follow TIdieR guidelines [89]. Secondly, more transparent and comprehensive descriptions of BCTs used, fidelity to intervention protocol and clarity regarding the theoretical constructs and models used in published studies is required.

From a practice perspective, findings of this manuscript suggest support for implementing a graded approach to gradually increasing frequency and intensity of intervention content, structuring interventions so that the key components are delivered by credible experts (i.e. exercise physiologists and dietitians) and alignment of behaviour change techniques to target behaviours following a comprehensive behavioural diagnosis.

## Conclusion

Our findings show that combined diet and physical activity interventions achieved clinically meaningful reductions in HbA_1c_ at 3 and 6 months, but these were not sustained at 12 and 24 months. We identified four BCTs and nine intervention features associated with reductions in HbA_1c_. These exploratory findings may guide future research into BCTs such as ‘instruction on how to perform a behaviour’, ‘behavioural practice/rehearsal’, ‘action planning’, and ‘demonstration of the behaviour’ which seemed to be associated with better outcomes in type 2 diabetic adults in addition to the intervention features identified.

## Additional files

Additional file 1:
**1.1**. PRISMA. **1.2**. Search strategy. **1.3**. BCT coding rubric/rules. **1.4**. Summary Table of included studies. **1.5**. Risk of bias assessment for included studies. **1.6**. Methodological quality and risk of bias of individual studies. **1.7**. Treatment fidelity. **1.8**. Meta-analyses of body weight changes at 3, 6, 12 and 24 months. **1.9**. Overall meta-analysis of body weight changes. **1.10**. Intervention content. **1.11**. Cohen’s kappa and PABAK for BCT Coding reliability. **1.12**. BCTs used in dietary aspect of intervention. **1.13**. BCTs used in physical activity aspect of intervention. **1.14**. Breakdown of frequency of BCTs used by Category for diet and physical activity behaviour. **1.15**. Breakdown of BCTs ‘NOT’ used by category and individual BCTs. **1.16**. Moderator analysis of diet BCTs. **1.17**. Moderator analysis of physical activity BCTs. (DOCX 328 kb)
Additional file 2: Excel file documenting text for each BCT for all included studies. (XLSX 113 kb)
