# Supplementary material for: Behaviour change techniques targeting both diet and physical activity in type 2 diabetes: A systematic review and meta-analysis
Source: Int J Behav Nutr Phys Act. 2017 Feb 8;14:18. doi: 10.1186/s12966-016-0436-0 (PMC5299734; doi:10.1186/s12966-016-0436-0)
Supplement: Additional file 1: — 1.1. PRISMA. 1.2. Search strategy. 1.3. BCT coding rubric/rules. 1.4. Summary Table of included studies. 1.5. Risk of bias assessment for included studies. 1.6. Methodological quality and risk of bias of individual studies. 1.7. Treatment fidelity. 1.8. Meta-analyses of body weight changes at 3, 6, 12 and 24 months. 1.9. Overall meta-analysis of body weight changes. 1.10. Intervention content. 1.11. Cohen’s kappa and PABAK for BCT Coding reliability. 1.12. BCTs used in dietary aspect of intervention. 1.13. BCTs used in physical activity aspect of intervention. 1.14. Breakdown of frequency of BCTs used by Category for diet and physical activity behaviour. 1.15. Breakdown of BCTs ‘NOT’ used by category and individual BCTs. 1.16. Moderator analysis of diet BCTs. 1.17. Moderator analysis of physical activity BCTs. (DOCX 328 kb) [file 12966_2016_436_MOESM1_ESM.docx]

**Additional File 1**

1.1. PRISMA

1.2. Search strategy

1.3. BCT coding rubric / rules

1.4. Summary Table of included studies

1.5. Risk of bias assessment for included studies

1.6. Methodological quality and risk of bias of individual studies

1.7. Treatment fidelity

1.8. Meta-analyses of body weight changes at 3, 6, 12 and 24 months

1.9. Overall meta-analysis of body weight changes

1.10. Intervention content

1.11. Cohen’s kappa and PABAK for BCT Coding reliability

1.12. BCTs used in dietary aspect of intervention

1.13. BCTs used in physical activity aspect of intervention

1.14. Breakdown of frequency of BCTs used by Category for diet and physical activity behaviour

1.15. Breakdown of BCTs ‘NOT’ used by category and individual BCTs

1.16. Moderator analysis of diet BCTs

1.17. Moderator analysis of physical activity BCTs

**Additional file 1.1 PRISMA Checklist**

| **Section/topic** | **#** | **Checklist item** | **Reported on page #** |
| --- | --- | --- | --- |
| **TITLE** | | |  |
| Title | 1 | Identify the report as a systematic review, meta-analysis, or both. | 1 |
| **ABSTRACT** | | |  |
| Structured summary | 2 | Provide a structured summary including, as applicable: background; objectives; data sources; study eligibility criteria, participants, and interventions; study appraisal and synthesis methods; results; limitations; conclusions and implications of key findings; systematic review registration number. | 2 |
| **INTRODUCTION** | | |  |
| Rationale | 3 | Describe the rationale for the review in the context of what is already known. | 3-4 |
| Objectives | 4 | Provide an explicit statement of questions being addressed with reference to participants, interventions, comparisons, outcomes, and study design (PICOS). | 4 |
| **METHODS** | | |  |
| Protocol and registration | 5 | Indicate if a review protocol exists, if and where it can be accessed (e.g., Web address), and, if available, provide registration information including registration number. | A protocol was followed but it was not published |
| Eligibility criteria | 6 | Specify study characteristics (e.g., PICOS, length of follow-up) and report characteristics (e.g., years considered, language, publication status) used as criteria for eligibility, giving rationale. | 5 |
| Information sources | 7 | Describe all information sources (e.g., databases with dates of coverage, contact with study authors to identify additional studies) in the search and date last searched. | 6 |
| Search | 8 | Present full electronic search strategy for at least one database, including any limits used, such that it could be repeated. | 6 and Suppl. file B1. |
| Study selection | 9 | State the process for selecting studies (i.e., screening, eligibility, included in systematic review, and, if applicable, included in the meta-analysis). | 6 |
| Data collection process | 10 | Describe method of data extraction from reports (e.g., piloted forms, independently, in duplicate) and any processes for obtaining and confirming data from investigators. | 6 |
| Data items | 11 | List and define all variables for which data were sought (e.g., PICOS, funding sources) and any assumptions and simplifications made. | 7-8 |
| Risk of bias in individual studies | 12 | Describe methods used for assessing risk of bias of individual studies (including specification of whether this was done at the study or outcome level), and how this information is to be used in any data synthesis. | 7 |
| Summary measures | 13 | State the principal summary measures (e.g., risk ratio, difference in means). | 8-9 |
| Synthesis of results | 14 | Describe the methods of handling data and combining results of studies, if done, including measures of consistency (e.g., I^2^) for each meta-analysis. | 8-9 |

| **Section/topic** | **#** | **Checklist item** | **Reported on page #** |
| --- | --- | --- | --- |
| Risk of bias across studies | 15 | Specify any assessment of risk of bias that may affect the cumulative evidence (e.g., publication bias, selective reporting within studies). | 7 |
| Additional analyses | 16 | Describe methods of additional analyses (e.g., sensitivity or subgroup analyses, meta-regression), if done, indicating which were pre-specified. | BCT coding 7, intervention features coding 8, meta-analysis 8, moderator analysis 9 |
| **RESULTS** | | |  |
| Study selection | 17 | Give numbers of studies screened, assessed for eligibility, and included in the review, with reasons for exclusions at each stage, ideally with a flow diagram. | 9 and Figure 1 (28) |
| Study characteristics | 18 | For each study, present characteristics for which data were extracted (e.g., study size, PICOS, follow-up period) and provide the citations. | 9 and Supplementary file A3 |
| Risk of bias within studies | 19 | Present data on risk of bias of each study and, if available, any outcome level assessment (see item 12). | 9-10, and Supplementary files A1 and B4 |
| Results of individual studies | 20 | For all outcomes considered (benefits or harms), present, for each study: (a) simple summary data for each intervention group (b) effect estimates and confidence intervals, ideally with a forest plot. | 9-13, Supplementary file A3 |
| Synthesis of results | 21 | Present results of each meta-analysis done, including confidence intervals and measures of consistency. | 12-13, 29 and Supplementary file A2, B12 and B13 |
| Risk of bias across studies | 22 | Present results of any assessment of risk of bias across studies (see Item 15). | 9-10, Supplementary files A1 and B4 |
| Additional analysis | 23 | Give results of additional analyses, if done (e.g., sensitivity or subgroup analyses, meta-regression [see Item 16]). | BCT Coding 10-11, Supplementary files A4, B6, B7, B8, B9, Fidelity assessment 9-10 and B5, Moderator Analysis 10-12, 30-31, Supplementary files A5, B10, B11 |
| **DISCUSSION** | | |  |
| Summary of evidence | 24 | Summarize the main findings including the strength of evidence for each main outcome; consider their relevance to key groups (e.g., healthcare providers, users, and policy makers). | 13-20 |
| Limitations | 25 | Discuss limitations at study and outcome level (e.g., risk of bias), and at review-level (e.g., incomplete retrieval of identified research, reporting bias). | 20-21 |
| Conclusions | 26 | Provide a general interpretation of the results in the context of other evidence, and implications for future research. | 21-22 |
| **FUNDING** | | |  |
| Funding | 27 | Describe sources of funding for the systematic review and other support (e.g., supply of data); role of funders for the systematic review. | 23 |

*From:*  Moher D, Liberati A, Tetzlaff J, Altman DG, The PRISMA Group (2009). Preferred Reporting Items for Systematic Reviews and Meta-Analyses: The PRISMA Statement. PLoS Med 6(6): e1000097. doi:10.1371/journal.pmed1000097

For more information, visit: **www.prisma-statement.org**.

Page 2 of 2

**Additional file 1.2 Search strategy and search terms**

| Search |  | ("type 2 diab*" OR "type II diab*" OR "non-insulin-dependent diabetes mellitus") |
| --- | --- | --- |
| All fields | AND | (diet OR nutrition OR exercise OR "physical activity" OR lifestyle OR "weight loss") |
| All fields | AND | (intervention OR behav* OR program* OR training) |
| All fields | AND | (random*) |
| All fields | AND | (treat* OR manag* OR "with type 2 diab*" OR "with type II diab*" OR "with non-insulin-dependent diabetes mellitus") |
| All fields | AND | ("random* control* trial" OR "random* clinical trial" OR "rct") |
| All fields | AND NOT | (cancer OR "gestational diabetes" OR "type 1 diabetes" OR child* OR adolescent OR prevent*) |
| Limit |  | English |
| Exclude |  | Conference paper, note, short survey, letter, editorial |
| Exclude |  | Reviews |

**Additional file 1.3 BCT Coding Rubric / Rules**

**Overall Coding Guidelines**

1. Read the whole text before starting to code for BCTs.

2. Only identify a BCT once, even if it appears several times in the text.

3. Identify the BCT in its most likely place, (identify article and page number).

4. Only include BCTs that pertain to the behaviour the intervention is trying to change.

5. Inferring the presence of a BCT is not sufficient information to code the BCT as present in the text, there must be clear evidence to indicate the presence of a BCT.

6. Before assigning a code, read the BCT label and description (including notes) in the taxonomy or using the app.

**Specific Coding Rules**

| **Code the following BCT** | **If this information is provided** |
| --- | --- |
| 1.1 Goal setting (behaviour): | “Goals of the intervention were modest weight loss (5% of initial weight) and dietary intake as well as physical activity reflecting national recommendations (20 –22) (Wolf et al 2004). If these goals are communicated to the person then code BCT 1.1 Goal setting (behaviour). |
| - 1. Goal setting (behaviour)   1.4 Action planning | “The programme focused on moderate weight loss with a goal of 25% of calories from dietary fat” (Mayer-Davis et al 2004). |
| 1.1 Goal setting (behaviour)  1.5 Review behaviour goal(s) | “To harness the benefits of peer support, subjects set weekly goals for specific changes in their eating behaviours to decrease portion sizes and make healthier food choices. These goals were shared with the group at the end of class, and progress was reported at the beginning of the next class” (Goldhaber-Fiebert et al 2003). |
| 1.3 Goal setting (outcome) | “This component included topics such as relapse prevention and weight maintenance and strategies such as goal setting” (Agurs-Collins et al 1997). According to the online BCT training: If goal unspecified or a behavioural outcome, code 1.3, Goal setting (outcome). |
| 1.4 Action Planning | “patients were advised to lower their calorie intake by 500 kcal/day. The patients were assigned to a low-carbohydrate diet developed by Ludwig [14] as modified by Worm [15]. Emphasis was placed on preference for low-GI carbohydrates, but not on avoidance of carbohydrates as required by the Atkins diet” (Luley et al 2011). |
| 1.4 Action Planning | “exercise and diet plus exercise groups were requested to walk briskly for 120 min every day, which corresponds to an energy expenditure of approximately 500 kcal ⁄ day” (Koo et al 2010). |
| 1.4 Action Planning | “asked to reduce their usual energy intake to 1200 kcal ⁄ day for weight reduction, dietary macronutrient composition was the same for all groups; namely, 50–55% of energy intake as carbohydrate, 15–20% as protein and 20–25% as fat” (Koo et al 2010). |
| 1.4 Action Planning | “Concerning exercise, the patients were advised to increase their usual daily physical activity, like walking or cycling, rather than to engage in particular sports. It was recommended to keep the pulse below 120/min and to perform the exercise slowly enough to be able to talk at the same time” (Luley et al 2011). |
| 1.8 Behavioural contract  1.4 Action planning  1.1 Goal setting (behaviour) | “Written contracts were used to identify goals and how, when, and where participants will modify their behaviours to achieve them” (Espeland et al 2007 LA). |
| 2.1 Monitoring of behaviour by others without feedback | **“**All subjects were requested to attach an accelerometer to their belts all day long during the intervention period. Data from the accelerometers were analysed using physical activity analysis software v1.0 [4] to determine energy expenditure” (Koo et al 2010). |
| 2.3 Self-monitoring of behaviour | Give patient a pedometer and a form for recording daily total number of steps. Note: if monitoring is part of a data collection procedure rather than a strategy aimed at changing behaviour, do not code (BCTs Taxonomy info 2.3). |
| 2.4 Self-monitoring of outcome(s) of behaviour | “they were weighed at each visit” This BCT (2.4) was coded for both physical activity and diet behaviour as weight change is the outcome for both of these behaviours (Agurs-Collins et al 1997). |
| 2.4 Self-monitoring of outcome(s) of behaviour | “Emphasized behaviour change strategies, these included the following: identifying the benefits of weight loss; setting goals for gradual changes to physical activity and dietary intake; self monitoring progress” This BCT was used because the specific behaviour was not identified but self monitoring did occur (Eakin et al 2014). |
| 3.3 Social support (emotional) | “The group setting provided for social interaction and peer support, and participants were encouraged to bring their spouses or significant others to the classes”. This BCT 3.3 was coded for both behaviours, as both diet and PA were interventions were carried out during the class (Agurs-Collins et al 1997). |
| 4.1 Instruction on how to perform a behaviour | “standard dietary and exercise advice after randomization and at the end of the study, with reviews by a study doctor and nurse at baseline and at 6 and 12 months” (Andrews et al 2011). |
| 4.1 Instruction on how to perform a behaviour  6.1 Demonstration of the behaviour  8.1 Behavioural practice / rehearsal | When the person attends classes such as exercise or cookery, i.e. “attended an exercise class”, code these three BCTs, 4.1, 6.1 and 8.1. |
| 6.1 Demonstration of the behaviour | “The dietary intervention consisted of 14 educational sessions with a registered dietitian. The dietary sessions covered a different subject regarding proper dietary management each week, such as general dietary management goals, using the food exchange table, eating out and snacking” (Kim et al 2014). |
| 9.1 Credible source | “The classes were taught by three nutritionists who were enrolled in a nutrition Master’s degree program at the University of San Jose” (Goldhaber-Fibert et al 2003). Credible source was only coded as a BCT where information was delivered by an expert in that area. |
| 9.2 Pros and cons | “Weigh the pros and cons of each option” (Look Ahead Trial), SP7, P 4*. |
| 12.1 Restructuring the physical environment | “suggestions include serving meals on a small plate to make the food appear bigger, avoiding eating a meal while watching television, and putting extra food away after serving oneself” (Goldhaber-Fibert et al 2003). |
| 12.3 Avoidance/reducing exposure to cues for the behaviour | “controlling or avoiding triggers to eat, and portion control” (Agurs-Collins et al 1997). |
| 12.5 Adding objects to the environment | This was also coded when a pedometer or accelerometer was used (Andrews et al 2011). Note use of a pedometer also indicates presence of BCT 2.3 ‘Self-monitoring of behaviour’. |

All BCTs coded and associated text providing rational for BCT is available from the author.

* For the Look Ahead Trial SP denotes Session plan 1-44, CM denotes Counselors manual 1-44.

**Do NOT Code**

| **Do Not Code the following BCT** | **If this information is provided** |
| --- | --- |
| 1.1 Goal setting (behaviour) | “Goals of the intervention were modest weight loss (5% of initial weight) and dietary intake as well as physical activity reflecting national recommendations (20 –22) (Wolf et al 2004). If these goals are NOT communicated to the person then do not code BCT 1.1 Goal setting (behaviour). |
| 1.4 Action planning | “Guidance was given on how many portions of each food group to choose and participants were specifically encouraged to choose foods in the lower ranges of energy density, fat content, and glycaemic index” (Andrews et al 2011). Encouragement vs. setting a goal action plan is different. |
| 1.4 Action planning | “The dietary modifications aimed to achieve an intake of 15% protein, 45%–50% carbohydrates, <35% fat per day, with a 1:1:1 ratio of polysaturated, monosaturated, and saturated fat, respectively” (Schultz et al 2011). If the person is not aware of this there is not enough evidence to code. If the person is aware of this, then we can code 1.4 Action planning. |
| 1.8 Behavioural contract | “Written contracts **may** be used to identify goals and how, when, and where participants will modify their behaviours to achieve them” (Espeland et al 2007 LA). The use of the word ‘may’ indicates that this was an option and was not definitely used. |
| 3.2 Social support (practical)  3.3 Social support (emotional) | “the group setting provided for social interaction and peer support, and participants were encouraged to bring their spouses or significant others to the classes” (Agurs-Collins et al 1997). It’s not specific if this is practical or emotional. |
| 4.1 Instruction on how to perform a behaviour | “Coaches conducted individual sessions that involved a health behaviour assessment and an education programme via computer”. This is a good example where there is not enough detail. The ‘Education’ program may have been about the ‘outcomes of PA’ rather than ‘instruction on how to perform the behaviour’. |
| 6.1 Demonstration of the behaviour  8.1 Behavioural practice / rehearsal | There is insufficent evidence to code based on the following text:  “a structured 16-session core cuniculum composed of behavioural strategies for weight loss and physical activity” (Mayer Davis et al 2004). |
| 8.2 Behaviour substitution | “suggestions include serving meals on a small plate to make the food appear bigger, avoiding eating a meal while watching television, and putting extra food away after serving oneself” (Goldhaber-Fibert et al 2003). The substitution needs to be explicit and a positive or neutral behaviour. For example, instead of eating while watching TV, participants were encouraged to do light exercises. |
| 10.1 Material incentive (behaviour) | “Monetary incentives were also used to enhance retention of subjects during the 12-month follow-up period. To encourage completion of standardized visits, research staff disclosed the incentive to subjects when scheduling visits by telephone”. These incentives were for retention not BCTs for changing PA/diet. They wanted them to stay in the study. (Mayer Davis et al 2004). |
| 10.2 Material reward (behaviour) | “Subjects received a $25 pharmacy gift certificate and refrigerator magnet with study logo at 3 months, a $20 grocery store gift certificate plus a study t-shirt at 6 months, and a $20 grocery store gift certificate plus a cookbook at 12 months”. These rewards were for retention not BCTs for changing PA/diet. They wanted them to stay in the study. (Mayer Davis et al 2004). |
| 13.5 Identity associated with changed behaviour | “To foster involvement and a sense of ownership and group identity, intervention participants in the first wave of classes (n = 25) were asked to develop a name for themselves” (Agurs-Collins et al 1997). This ‘technique’ is about creating a group dynamic not having someone ‘identify as an exerciser’ or as a healthy eater. |

**Additional file 1.4 Summary Table of included studies**

| Study ID (Reference number) | Setting | Country | Number of participants | Age [mean years (SD)] | Sex (% female) | Duration of diabetes [mean years (SD)] | HbA_1c_ baseline [mean % (SD)] | Body mass [mean kg (SD)] | Ethnic groups Caucasian (%) | Duration of intervention |
| --- | --- | --- | --- | --- | --- | --- | --- | --- | --- | --- |
| Agurs-Collins *et al.* 1997 | Hospital | USA | 64 | I: 62.4 (5.9)  C: 61 (5.7) | I: 66  C: 88 | I: NR  C: | I: 11 (1.7)  C: 10 (1.9) | I: 93.3 (18.6)  C: 94.9 (20.1) | I: 0  C: 0 | 6 months |
| Andrews *et al.* 2011 | National health trust | UK | 345** | I: 60 (9.7)  C: 59.5 (11.1) | I: 34  C: 37 | I: 0.53  C: 0.51 | I: 6.69 (0.99)  C: 6.72 (1.02) | I: 91.1 (16.9)  C: 93.9 (19) | I: 94  C: 97 | 12 months |
| Eakin *et al.* 2014 | Primary care | Australia | 302 | I: 57.7 (8.1)  C: 58.3 (9) | I: 44.4  C: 43 | I: 4*  C: 5* | I: 7.33 (1.5)  C: 7.33 (1.7) | I: 94.5 (18.7)  C: 95.3 (20.1) | I: 86.8  C: 88.1 | 18 months |
| Espeland *et al.* 2007 | 16 Study centers | USA | 5,145 | I: 58.6 (6.8)  C: 58.9 (6.9) | I: 59.3  C: 59.6 | I: 6.8 (6.5)  C: 6.8 (6.5) | I: 7.25 (0.72)  C: 7.31 (0.72) | I: 100.5 (19.6)  C: 100.8 (18.8) | I: 63.1  C: 63.3 | 10 years |
| Goldhaber-Fibert *et al.* 2003 | Community centers | USA | 75 | I: 60 (10)  C: 57 (9) | I: 82.5  C: 74.3 | I: NR  C: | I: 8.6 (3.7)  C: 8.6 (3.9) | I: 72.4 (14.6)  C: 71.9 (12.4) | I: NR  C: | 12 weeks |
| Kim *et al.* 2006 | Outpatient clinic | Korea | 58 | I: 55 (8.1)  C: 53.8 (9) | I: 81.25  C: 69.2 | I: 7.9 (6.5)  C: 10 (6.6) | I: 8.5 (1.4)  C: 8.6 (1.3) | I: 65.7 (13.5)  C: 66.6 (13.9) | I: NR  C: | 6 months |
| Kim *et al.* 2014 | Outpatient clinic | Korea | 35 | I: 48.4 (8.6)  C: 48.3 (8.2) | I: 50  C: 41.2 | I: 5.4 (3.4)  C: 4.5 (3.6) | I: 7.5 (0.7)  C: 7.7 (0.7) | I: 78.3 (14.8)  C: 76.3 (11.4) | I: NR  C: | 12 weeks |
| Koo *et al.* 2010 | Hospital | Korea | 32 | I: 53 (8)  C: 57 (8) | I: 100  C: 100 | I: 7 (7)  C: 8 (6) | I: 8 (1.8)  C: 7.5 (1.1) | I: 69.4  C: 66 | I: NR  C: | 12 weeks |
| Luley *et al*. 2011 | Clinic | Germany | 68 | I: 57 (9)  C: 58 (7) | I: 57  C: 46 | I: NR  C: | I: 7.5 (1.1)  C: 7.6 (1.1) | I: 102.1 (20)  C: 101.4 (17) | I: NR  C: | 6 months |
| Mayer-Davis *et al.* 2004 | Primary health care center | USA | 105** | I: 59.7 (8.6)  C: 62.4 (9.5) | I: 78  C: 79 | I: 8.4 (6.5)  C: 12.7 (10.6) | I: 10.2 (2.5)  C: 9.6 (2.9) | I: 99.5 (17.1)  C: 93 (20.3) | I: 14.3  C: 26.8 | 12 months |
| Schultz *et al.* 2011 | Hospital | Australia | 185 | I: 54.7 (11.3)  C: 53.8 (8.1) | I: 49  C: 39 | I: 5.8 (6.4)  C: 5.9 (5.8) | I: 7.52 (1.5)  C: 7.54 (1.43) | I: NR  C: | I: NR  C: | 12 months |
| Vanninen *et al.* 1992 | Outpatient clinic | Finland | 78 | I: NR  C: | I: 44.7  C: 40 | I: NR  C: | I: 7.1 (1.5)  C: 7.7 (2.05) | I: NR  C: | I: NR  C: | 12 months |
| Wolf *et al.* 2004 | University health system | USA | 147 | I: 53.3 (8.6)  C: 53.4 (8.0) | I: 62  C: 58 | NR | I: 7.9 (1.6)  C: 7.5 (1.5) | I: 107.1 (25.5)  C: 106.7 (24.3) | I: 85  C: 74 | 12 months |

***** Median figure reported

****** Number of subjects reported is for control group and diet and exercise groups only, other group is not included

**Additional file 1.5 Risk of bias assessment for included studies**


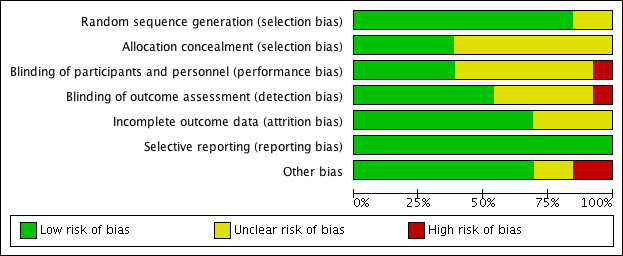


**
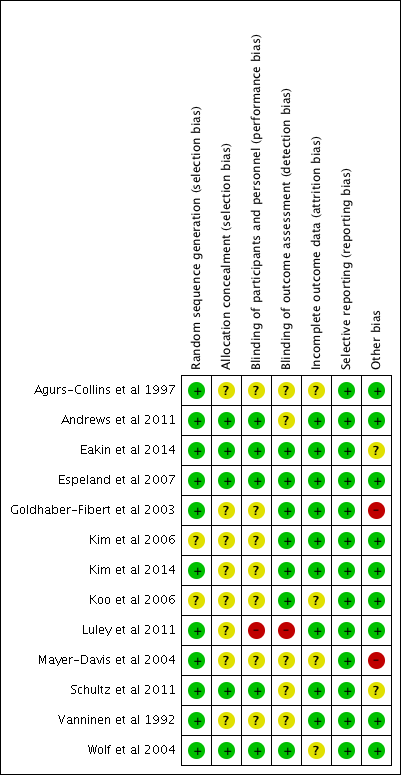
**

**Additional file 1.6 Methodological quality and risk of bias of individual studies**

| **Study ID** | **Power calculation (sample size achieved at final follow-up)** | **Attrition rate** | **Intention to treat** | **A** | **B** | **C** | **D** | **E** | **F** | **Study free from other biases** | **Notes / Comments** |
| --- | --- | --- | --- | --- | --- | --- | --- | --- | --- | --- | --- |
| Agurs-Collins et al 1997 | yes/no | 9, 14.1% | NR | low | unclear | unclear | unclear | unclear | low | low | Intention to treat NR but we think they did use ITT because all participants were included regardless of how many sessions they completed |
| Andrews et al 2011 | yes/yes | 14, 2.4% | yes | low | low | low | unclear | low | low | low |  |
| Eakin et al 2014 | yes/yes | 71, 23.5% | yes | low | low | low | Low | low | low | unclear | Compliance to the intervention protocol was low, therefore G 'Study free from other biases' is marked as 'unclear' |
| Espeland 2007 | yes/yes | 186, 3.6% | NR | low | low | low | Low | low | low | low | Intention to treat NR but we think they did use ITT |
| Goldhaber-Fiebert et al 2003 | yes/yes | 14, 18.7% | yes | low | unclear | unclear | Low | low | low | high | G 'study free from other bias' = High because so many people excluded from walking aspect of intervention |
| Kim et al 2006 | NR (NR) | NR | NR | unclear | unclear | unclear | Low | low | low | low | Attrition rate not explicitly reported but appears to be 0%, intention to treat NR but it appears to have 100% compliance |
| Kim et al 2014 | yes/yes | 3, 8.6% | NR | low | unclear | unclear | Low | low | low | low | Intention to treat NR but we think they did use ITT because all participants were included regardless of how many sessions they completed |
| Koo et al 2010 | NR(NR) | 6, 8.6% | No | unclear | unclear | unclear | Low | unclear | low | low | Intention to treat: No (p. 1089--Authors excluded people from analysis based on compliance), E: 'Incomplete outcome data' = unclear, but it might be high |
| Luley et al 2011 | unclear | 2, 2.9% | NR | low | unclear | high | high | low | low | low | Intention to treat NR, but we think they did use ITT because all participants were included regardless of how many sessions they completed. C and D 'blinding of participants and personnel', and 'blinding of outcome assessment' we think it might be high--p.287 says that the study authors reviewed weekly subject progress and sent reports. It’s also not clear that the authors calculated the power calculation a priori. |
| Mayer Davis et al 2004 | yes/no | 35, 18.7% | NR | low | unclear | unclear | unclear | unclear | low | High | Intention to treat NR but yes (they also reported the analysis only with compliant participants). Large difference between baseline HbA1c for intervention and control subjects, and a large reduction in HbA1c for control groups at 12 months |
| Schultz et al 2011 | yes/yes | 38, 17% | NR | low | low | unclear | Low | low | low | unclear | G Study free from other bias = 'unclear' due to baseline differences in fitness |
| Vanninen et al 1992 | NR/NR | 12, 15.4% | NR | low | unclear | unclear | unclear | low | low | low | Intention to treat NR but we think they did use ITT because all participants were included regardless of how many sessions they completed |
| Wolf et al 2004 | unclear | 29, 19.7% | yes | low | low | low | Low | unclear | low | low | It’s not clear that the authors calculated sample size a priori. |

**Additional file 1.7 Treatment fidelity strategies**

| **Study ID / Authors** | **Year** | **Design of study** | | | **Monitoring and improving provider training** | | | | **Monitoring and improving delivery of treatment** | | | | **Monitoring and improving receipt of treatment** | | | **Monitoring and improving enactment of treatment skills** | |
| --- | --- | --- | --- | --- | --- | --- | --- | --- | --- | --- | --- | --- | --- | --- | --- | --- | --- |
|  |  |  |  |  |  |  |  |  |  |  |  |  |  |  |  |  |  |
|  |  | **A** | **B** | **C** | **D** | **E** | **F** | **G** | **H** | **I** | **J** | **K** | **L** | **M** | **N** | **O** | **P** |
| Andrews et al* | 2011 | Yes | Yes | No | No | No | No | No | No | No | Unclear | No | No | No | No | No | Yes |
| Eakin et al* | 2014 | Unclear | No | No | Yes | Yes | Yes | Yes | No | Yes | Yes | Unclear | Unclear | No | No | No | Yes |
| Kim et al | 2006 | No | No | No | Unclear | No | No | No | No | No | No | No | No | No | No | No | Yes |
| Koo et al | 2010 | No | Unclear | No | No | No | No | No | No | No | No | No | No | No | No | No | Yes |
| Luley et al | 2011 | No | No | No | No | No | No | No | No | No | No | No | No | No | No | No | Unclear |
| Mayer Davis et al* | 2004 | No | No | No | Yes | Yes | Yes | Unclear | No | Unclear | No | No | No | No | No | No | Yes |
| Espeland et al* | 2007 | Yes | No | Unclear | Yes | Yes | Yes | Unclear | No | Yes | Yes | Unclear | No | No | No | No | Yes |
| Wolf et al | 2004 | No | No | No | No | No | No | No | No | No | No | No | Unclear | No | No | No | No |
| Goldhaber-Fiebert et al ** | 2003 | No | No | No | Yes | Yes | No | No | No | No | No | No | Unclear | No | No | No | Yes |
| Agurs-Collins et al | 1997 | Unclear | No | No | No | No | No | No | No | No | No | No | Unclear | No | No | No | Yes |
| Vanninen et al | 1992 | No | No | No | No | No | No | No | No | No | No | No | No | No | No | No | Yes |
| Kim et al | 2014 | No | No | No | No | No | No | No | No | No | No | No | No | No | No | No | Yes |
| Schultz et al* | 2011 | No | No | No | No | No | No | No | No | No | No | No | No | No | No | No | Yes |

* Information sought from extra files

** Goldhaber-Fibert et al 2003, Design of study reported but only 9 out of 40 subjects in intervention group participated in exercise aspect of intervention

Yes = a treatment fidelity strategy was reported and described; Unclear = insufficient information to make a judgment about the presence of absence of treatment fidelity strategy; No = treatment fidelity strategy was not reported

**Subcategories**

**Design of study**

A Ensure same treatment dose within condition

B Ensure equivalent dose across condition

C Plan for implementation setbacks

**Monitoring and improving provider training**

D Standardise training

E Ensure provider skill acquisition

F Minimize "drift" in provider skills.

G Accommodate provider differences

**Monitoring and improving delivery of treatment**

H Control for provider differences

I Reduce differences within treatment

J Ensure adherence to treatment protocol

K Minimise contamination between conditions

**Monitoring and improving receipt of treatment**

L Ensure participant comprehension

M Ensure participant ability to use cognitive skills

N Ensure participant ability to perform behavioral skills

**Monitoring and improving enactment of treatment skills**

O Ensure participant use of cognitive skills

P Ensure participant use of behavioural skills

**Additional file 1.8** **Meta-analysis of body weight changes at 3, 6, 12 and 24 months**

A Difference in body weight at 3 months


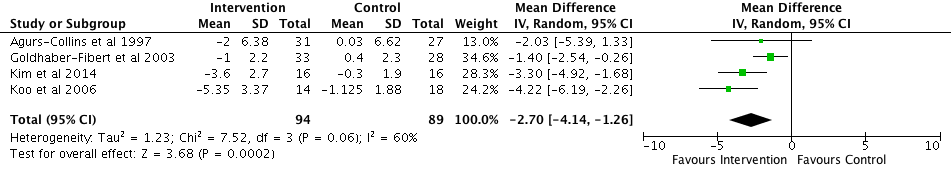


B Difference in body weight at 6 months


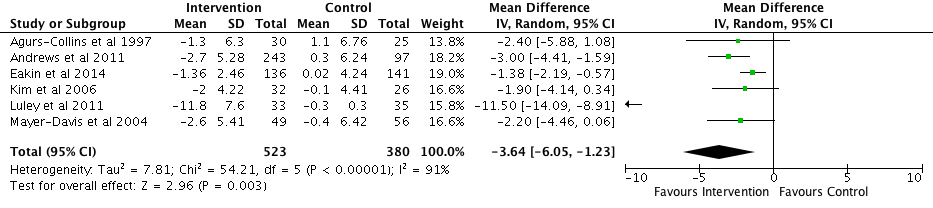


C Difference in body weight at 12 months


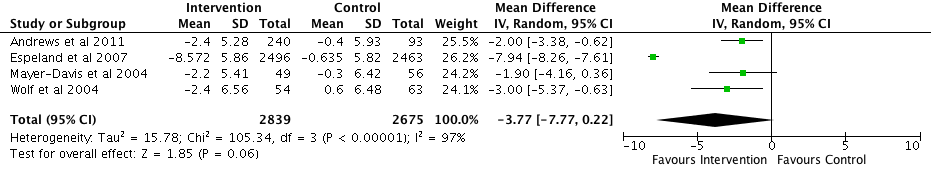


D Difference in body weight at 24 months


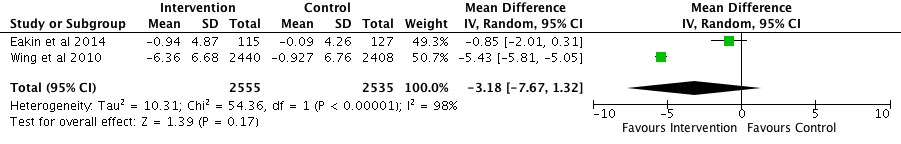


**Additional file 1.9 Overall meta-analysis of body weight changes**


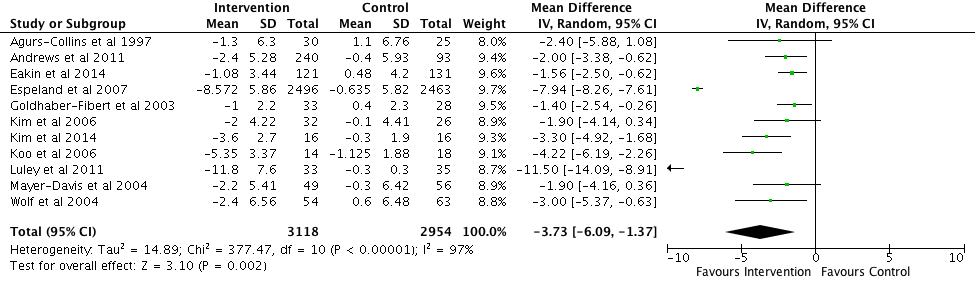


Meta analysis of mean difference in body weight (kg) from baseline (studies with multiple time points are represented by time point closest to the end of intervention)

**Additional file 1.10 Intervention Content**

| **Study ID** | **Diet** | **Physical Activity** | **Intensity** | **Duration** |
| --- | --- | --- | --- | --- |
| Agurs-Collins et al 1997 | Culturally adapted diet, 55-60% carbohydrate, 12-20% protein, <30% from fat (weight loss target was at least 4.5kg) | Moderate low impact aerobic physical activity 3 days per week | 12 weekly sessions for 1st 3 months in groups, plus 1 individual diet session, 6 biweekly sessions for following three months | 6 months |
| Andrews et al 2011 | Intensive diet aimed at 5-10% loss in body weight, not prescriptive, low energy dense, low fat, and low glycaemic index, following Diabetes UK Dietary guidelines. | 30 min of brisk walking (pedometer based) on at least 5 days per week in addition to current PA levels. | Participants met dietitian for 1 hour at randomization and 30 min at 3, 6, 9 and 12 months, plus 9 x 30 min appointments with study nurse | 12 months |
| Eakin et al 2014 | Individualized advice aimed to reduce intake by 500kcal, healthy eating principles and low fat diet (target weight loss of 5-10%) | Target of 210 min/week of moderate intensity aerobic exercise, plus resistance exercise, 2-3 sessions per week. | Workbook and up to 27 telephone calls (4 weekly calls, fortnightly for 5 months, and monthly for 12 months) | 18 months |
| Espeland 2007  (Look Ahead Study) | Individualized caloric restriction and meal replacement based on initial weight (target of >10% weight loss) | Gradual progression toward a goal of 175 min of moderate intensity exercise per week (home based) | Subject seen weekly for the first 6 months, 3 times per month for the next six months (group and individual meetings). In 2 through to 4 years, subjects were seen individually once per month and contacted each month in addition to meeting. | 11 Years, (data reported here for 2 years) |
| Goldhaber-Fiebert et al 2003 | Community based nutrition intervention focused on portion control, weight reduction and use of healthier food substitutes | 60 min walking group 3 times per week | 11 weekly nutrition classes (90 min each), 60 min supervised walking group 3 times per week | 12 months |
| Kim et al 2006 | Recommended dietary intake aimed at 5% weight loss | Moderate intensity, such as brisk walking, for at least 150 min/wk | 1 lesson each week for 16 weeks, monthly sessions for the remainder of the 6 months | 6 months |
| Kim et al 2014 | Calorie reduction of 500 kcal/day, 55-60% carbohydrate, 15-20% protein, 20-25% fat (target of 7% weight loss) | Aerobic (50-70% of max heart rate for 30-40 min) and resistance training programme 3 times a week (50% 1RM) | Exercise & resistance training 3 times per week for 12 weeks, diet: 14 sessions with dietitian | 12 weeks |
| Koo et al 2010 | Reduce energy intake to 1200kcal / day, and individual education based on 3 day diary every 2 weeks, 50-55% carbohydrate, 15-20% protein, 20-25% fat | 120 min of brisk walking every day (accelerometer used) | Education based intervention, subjects met with interventionists every 2 weeks for 12 weeks | 12 weeks |
| Luley et al 2011 | Low calorie, low carbohydrate, low GI diet (reduction of 500kcal/day) | Increase daily physical activity using telemonitoring (maintain heart rate below 120 beats per min) | Once per week for 4 weeks, followed by once every 4 weeks for blood sampling for 6 months | 6 months |
| Mayer Davis et al 2004 | Culturally appropriate diet intervention, 25% of calories from dietary fat, low fat, low calorie diet based on DPP* | 150 minutes of physical activity per week | Subjects met with Nutritionist weekly for the first 4 months, fortnightly for the next 2 months, and once a month for the remaining 6 months | 12 months |
| Schultz et al 2011 | Individualized diet aimed at 7% decrease in body weight, 45-50% carbohydrate, <35% fat, 15% protein | Supervised aerobic and resistance training. Minimum of 150 min/wk of at least moderate intensity aerobic exercise plus 2 gym based training sessions of moderate intensity | 4 weeks of 2 sessions of supervised training (1 hour each) + 30 mins at home, next 11 months were home based with weekly telephone follow up, dietitians were met each month | 12 months |
| Vanninen et al 1992 | Goals were energy restriction, restriction of intake of fat, moderate intake of complex carbohydrates | Increase physical activity to 30-60 minutes 3-4 times a week (recommended heart rate was 110-140 beats per min) | 3 month basic education, 6 visits in total to outpatient clinic, every 2 months for 12 months | 12 months |
| Wolf et al 2004 | Goals of intervention were modest weight loss (5%) based on national diabetes guidelines and recommendations (low calorie diet of 500-100kcal reduction) | Based on national diabetes guidelines and recommendations, 30-40 mins of moderate physical activity 3-5 days per week | 6 x individual sessions during year, 6 x group sessions during year, monthly telephone follow up | 12 months |

- DPP Diabetes prevention programme 2002,

**Additional file 1.11 Cohen’s kappa and PABAK**

| **Study ID** | **BCTs** | **Agree present** | **Coder 1 Present; Coder 2 Absent** | **Coder 2 Present; Coder 1 Absent** | **Agree absent** | **Kappa** | **PABAK** |
| --- | --- | --- | --- | --- | --- | --- | --- |
| Luley et al 2011 | Diet | 6 | 2 (0) | 1 (0) | 84 | 0.78 | 0.94 |
| Kim et al 2006 | Diet | 2 | 4 (0) | 1 (0) | 86 | 0.82 | 0.96 |
| Kim et al 2014 | Diet | 8 | 1 (0) | 0 (0) | 84 | 0.94 | 0.98 |
| Koo et al 2010 | Diet | 4 | 2 (0) | 0 (0) | 87 | 0.79 | 0.96 |
| Mayer Davis et al 2004 | Diet | 10 | 2 (0) | 0 (0) | 81 | 0.9 | 0.96 |
| Vanninen et al 1992 | Diet | 3 | 3 (0) | 1 (0) | 86 | 0.58 | 0.91 |
| Agurs Collins et al 1997 | Diet | 10 | 6 (0) | 3 (0) | 74 | 0.63 | 0.81 |
| Wolf et al 2004 | Diet | 6 | 1 (0) | 1 (0) | 85 | 0.85 | 0.96 |
| Goldhaber-Fibert et al 2003 | Diet | 10 | 3 (0) | 1 (0) | 79 | 0.81 | 0.91 |
| Andrews et al 2011 | Diet | 10 | 1 (0) | 1 (0) | 81 | 0.9 | 0.96 |
| Schultz et al 2011 | Diet | 6 | 2 (0) | 4 (0) | 81 | 0.63 | 0.87 |
| Eakin et al 2014 | Diet | 15 | 5 (0) | 0 (0) | 73 | 0.82 | 0.89 |
| Espeland et al 2007 | Diet | 7 | 1 (0) | 3 (0) | 82 | 0.75 | 0.91 |
| Espeland et al 2007* | Diet | 13 | 3 (0) | 2 (0) | 75 | 0.81 | 0.89 |
| **AVERAGE** |  |  |  |  |  | **0.79** | **0.92** |
|  |  |  |  |  |  |  |  |
| Luley et al 2011 | Physical Activity | 6 | 4 (0) | 0 (0) | 83 | 0.73 | 0.91 |
| Kim et al 2006 | Physical Activity | 8 | 1 (0) | 0 (0) | 83 | 0.94 | 0.98 |
| Kim et al 2014 | Physical Activity | 9 | 1 (0) | 0 (0) | 83 | 0.94 | 0.98 |
| Koo et al 2010 | Physical Activity | 7 | 0 (0) | 0 (0) | 86 | 1 | 1 |
| Mayer Davis et al 2004 | Physical Activity | 7 | 4 (0) | 0 (0) | 80 | 0.76 | 0.91 |
| Vanninen et al 1992 | Physical Activity | 4 | 2 (0) | 1 (0) | 86 | 0.71 | 0.94 |
| Agurs Collins et al 1997 | Physical Activity | 8 | 6 (0) | 3 (0) | 76 | 0.59 | 0.81 |
| Wolf et al 2004 | Physical Activity | 3 | 1 (0) | 3 (0) | 86 | 0.58 | 0.91 |
| Goldhaber-Fibert et al 2003 | Physical Activity | 4 | 3 (0) | 1 (0) | 85 | 0.64 | 0.91 |
| Andrews et al 2011 | Physical Activity | 8 | 1 (0) | 0 (0) | 82 | 0.94 | 0.98 |
| Schultz et al 2011 | Physical Activity | 7 | 1 (0) | 4 (0) | 81 | 0.71 | 0.89 |
| Eakin et al 2014 | Physical Activity | 17 | 5 (0) | 1 (0) | 70 | 0.81 | 0.87 |
| Espeland et al 2007 | Physical Activity | 6 | 1 (0) | 4 (0) | 82 | 0.68 | 0.89 |
| Espeland et al 2007* | Physical Activity | 15 | 2 (0) | 2 (0) | 74 | 0.86 | 0.91 |
| **AVERAGE** |  |  |  |  |  | **0.78** | **0.92** |

* The Espeland et al. 2007 article plus the method paper Wadden et al. 2006

Note: For the 88 supporting documents available from the Look Ahead Website, Coder 1 (KC) coded all available information, Coder 2 (LQ) checked the results and the master coder (HG) arbitrated if any disagreements arose.

**Additional file 1.12 BCTs used in dietary aspect of intervention**

| **BCT no.** | **BCT Label Diet** | **(1)** | **(2)** | **(3)** | **(4)** | **(5)** | **(6)** | **(7)** | **(8)** | **(9)** | **(10)** | **(11)** | **(12)** | **(13)** | **Total** |
| --- | --- | --- | --- | --- | --- | --- | --- | --- | --- | --- | --- | --- | --- | --- | --- |
| **4.1** | Instruction on how to perform a behaviour | \|  \| \| --- \| | \|  \| \| --- \| |  | \|  \| \| --- \| | \|  \| \| --- \| | \|  \| \| --- \| | \|  \| \| --- \| | \|  \| \| --- \| | \|  \| \| --- \| | \|  \| \| --- \| | \|  \| \| --- \| | \|  \| \| --- \| | \|  \| \| --- \| | 12 |
| **9.1** | Credible source | \|  \| \| --- \| | \|  \| \| --- \| | \|  \| \| --- \| | \|  \| \| --- \| | \|  \| \| --- \| | \|  \| \| --- \| | \|  \| \| --- \| |  |  | \|  \| \| --- \| | \|  \| \| --- \| | \|  \| \| --- \| | \|  \| \| --- \| | 11 |
| **1.3** | Goal setting (outcome) | \|  \| \| --- \| | \|  \| \| --- \| | \|  \| \| --- \| | \|  \| \| --- \| |  | \|  \| \| --- \| | \|  \| \| --- \| |  |  | \|  \| \| --- \| | \|  \| \| --- \| | \|  \| \| --- \| | \|  \| \| --- \| | 10 |
| **1.1** | Goal setting (behaviour) | \|  \| \| --- \| | \|  \| \| --- \| | \|  \| \| --- \| | \|  \| \| --- \| | \|  \| \| --- \| | \|  \| \| --- \| |  | \|  \| \| --- \| | \|  \| \| --- \| | \|  \| \| --- \| |  |  |  | 9 |
| **1.4** | Action planning | \|  \| \| --- \| | \|  \| \| --- \| | \|  \| \| --- \| | \|  \| \| --- \| |  |  | \|  \| \| --- \| | \|  \| \| --- \| | \|  \| \| --- \| |  | \|  \| \| --- \| | \|  \| \| --- \| |  | 9 |
| **2.3** | Self-monitoring of behaviour | \|  \| \| --- \| | \|  \| \| --- \| | \|  \| \| --- \| | \|  \| \| --- \| | \|  \| \| --- \| | \|  \| \| --- \| | \|  \| \| --- \| | \|  \| \| --- \| |  | \|  \| \| --- \| |  |  |  | 9 |
| **3.1** | Social support (unspecified) | \|  \| \| --- \| | \|  \| \| --- \| | \|  \| \| --- \| | \|  \| \| --- \| | \|  \| \| --- \| |  |  |  | \|  \| \| --- \| | \|  \| \| --- \| |  |  | \|  \| \| --- \| | 8 |
| **2.2** | Feedback on behaviour | \|  \| \| --- \| |  | \|  \| \| --- \| | \|  \| \| --- \| |  |  | \|  \| \| --- \| | \|  \| \| --- \| |  |  | \|  \| \| --- \| |  |  | 6 |
| **6.1** | Demonstration of the behaviour | \|  \| \| --- \| |  |  | \|  \| \| --- \| | \|  \| \| --- \| |  | \|  \| \| --- \| |  | \|  \| \| --- \| |  |  |  |  | 5 |
| **1.2** | Problem solving | \|  \| \| --- \| |  | \|  \| \| --- \| | \|  \| \| --- \| |  |  |  |  |  | \|  \| \| --- \| |  |  |  | 4 |
| **2.5** | Monitoring outcome(s) of behaviour by others without feedback | \|  \| \| --- \| | \|  \| \| --- \| |  | \|  \| \| --- \| |  |  |  |  |  |  |  |  | \|  \| \| --- \| | 4 |
| **12.3** | Avoidance/reducing exposure to cues for the behaviour | \|  \| \| --- \| |  | \|  \| \| --- \| | \|  \| \| --- \| | \|  \| \| --- \| |  |  |  |  |  |  |  |  | 4 |
| **12.5** | Adding objects to the environment |  |  | \|  \| \| --- \| | \|  \| \| --- \| | \|  \| \| --- \| |  |  |  | \|  \| \| --- \| |  |  |  |  | 4 |
| **1.5** | Review behaviour goal(s) |  |  |  | \|  \| \| --- \| |  |  | \|  \| \| --- \| |  |  |  | \|  \| \| --- \| |  |  | 3 |
| **1.7** | Review outcome goal(s) |  | \|  \| \| --- \| |  | \|  \| \| --- \| |  |  |  |  | \|  \| \| --- \| |  |  |  |  | 3 |
| **2.4** | Self-monitoring of outcome(s) of behaviour |  | \|  \| \| --- \| | \|  \| \| --- \| | \|  \| \| --- \| |  |  |  |  |  |  |  |  |  | 3 |
| **2.7** | Feedback on outcome(s) of behaviour |  |  | \|  \| \| --- \| | \|  \| \| --- \| |  |  |  |  | \|  \| \| --- \| |  |  |  |  | 3 |
| **8.1** | Behavioural practice/rehearsal | \|  \| \| --- \| |  |  | \|  \| \| --- \| | \|  \| \| --- \| |  |  |  |  |  |  |  |  | 3 |
| **12.1** | Restructuring the physical environment |  |  | \|  \| \| --- \| | \|  \| \| --- \| | \|  \| \| --- \| |  |  |  |  |  |  |  |  | 3 |
| **3.3** | Social support (emotional) |  |  | \|  \| \| --- \| |  | \|  \| \| --- \| |  |  |  |  |  |  |  |  | 2 |
| **7.1** | Prompts/cues |  |  | \|  \| \| --- \| | \|  \| \| --- \| |  |  |  |  |  |  |  |  |  | 2 |
| **8.7** | Graded tasks | \|  \| \| --- \| |  | \|  \| \| --- \| |  |  |  |  |  |  |  |  |  |  | 2 |
| **10.3** | Non-specific reward |  |  | \|  \| \| --- \| | \|  \| \| --- \| |  |  |  |  |  |  |  |  |  | 2 |
| **10.9** | Self-reward |  |  | \|  \| \| --- \| | \|  \| \| --- \| |  |  |  |  |  |  |  |  |  | 2 |
| **15.4** | Self-talk |  |  | \|  \| \| --- \| | \|  \| \| --- \| |  |  |  |  |  |  |  |  |  | 2 |
| **8.2** | Behaviour substitution |  |  |  |  |  |  |  |  |  | \|  \| \| --- \| |  |  |  | 1 |
| **1.6** | Discrepancy between current behaviour and goal |  |  | \|  \| \| --- \| |  |  |  |  |  |  |  |  |  |  | 1 |
| **2.1** | Monitoring of behaviour by others without feedback |  |  |  |  |  |  |  | \|  \| \| --- \| |  |  |  |  |  | 1 |
| **3.2** | Social support (practical) |  |  |  | \|  \| \| --- \| |  |  |  |  |  |  |  |  |  | 1 |
| **5.1** | Information about health consequences |  |  |  | \|  \| \| --- \| |  |  |  |  |  |  |  |  |  | 1 |
| **6.2** | Social comparison |  |  |  |  | \|  \| \| --- \| |  |  |  |  |  |  |  |  | 1 |
| **7.5** | Remove aversive stimulus |  |  |  | \|  \| \| --- \| |  |  |  |  |  |  |  |  |  | 1 |
| **8.3** | Habit formation |  |  |  | \|  \| \| --- \| |  |  |  |  |  |  |  |  |  | 1 |
| **8.6** | Generalization of a target behaviour |  |  |  |  |  |  |  |  |  |  | \|  \| \| --- \| |  |  | 1 |
| **9.2** | Pros and cons |  |  |  | \|  \| \| --- \| |  |  |  |  |  |  |  |  |  | 1 |
| **10.2** | Material reward (behaviour) |  |  |  | \|  \| \| --- \| |  |  |  |  |  |  |  |  |  | 1 |
| **10.4** | Social reward |  |  |  | \|  \| \| --- \| |  |  |  |  |  |  |  |  |  | 1 |
| **10.6** | Non-specific incentive |  |  |  | \|  \| \| --- \| |  |  |  |  |  |  |  |  |  | 1 |
| **10.7** | Self-incentive |  |  |  | \|  \| \| --- \| |  |  |  |  |  |  |  |  |  | 1 |
| **11.2** | Reduce negative emotions |  |  |  | \|  \| \| --- \| |  |  |  |  |  |  |  |  |  | 1 |
| **12.2** | Restructuring the social environment |  |  |  | \|  \| \| --- \| |  |  |  |  |  |  |  |  |  | 1 |
| **13.1** | Identification of self as role model |  |  |  | \|  \| \| --- \| |  |  |  |  |  |  |  |  |  | 1 |
| **13.2** | Framing/reframing |  |  |  | \|  \| \| --- \| |  |  |  |  |  |  |  |  |  | 1 |
| **15.1** | Verbal persuasion about capability |  |  |  | \|  \| \| --- \| |  |  |  |  |  |  |  |  |  | 1 |
| **15.3** | Focus on past success |  |  |  | \|  \| \| --- \| |  |  |  |  |  |  |  |  |  | 1 |

**Key:** Studies are listed in alphabetical order (1) Agurs Collins et al., (2) Andrews et al., (3) Eakin et al., (4) Espeland et al., (5) Golhaber Fibert et al., (6) Kim et al 2006., (7) Kim et al 2014., (8) Koo et al., (9) Luley et al., (10) Mayer-Davis et al., (11) Schultz et al., (12) Vanninen et al., (13) Wolf et al.

**Additional file 1.13 BCTs used in physical activity aspect of intervention**

| **BCT no.** | **BCT Label Physical activity** | **(1)** | **(2)** | **(3)** | **(4)** | **(5)** | **(6)** | **(7)** | **(8)** | **(9)** | **(10)** | **(11)** | **(12)** | **(13)** | **Total** |
| --- | --- | --- | --- | --- | --- | --- | --- | --- | --- | --- | --- | --- | --- | --- | --- |
| **4.1** | Instruction on how to perform a behaviour | \|  \| \| --- \| |  | \|  \| \| --- \| | \|  \| \| --- \| | \|  \| \| --- \| | \|  \| \| --- \| | \|  \| \| --- \| | \|  \| \| --- \| | \|  \| \| --- \| | \|  \| \| --- \| | \|  \| \| --- \| | \|  \| \| --- \| | \|  \| \| --- \| | 12 |
| **1.1** | Goal setting (behaviour) | \|  \| \| --- \| | \|  \| \| --- \| | \|  \| \| --- \| | \|  \| \| --- \| | \|  \| \| --- \| | \|  \| \| --- \| |  | \|  \| \| --- \| | \|  \| \| --- \| | \|  \| \| --- \| | \|  \| \| --- \| | \|  \| \| --- \| |  | 11 |
| **1.4** | Action planning |  | \|  \| \| --- \| | \|  \| \| --- \| | \|  \| \| --- \| | \|  \| \| --- \| | \|  \| \| --- \| | \|  \| \| --- \| | \|  \| \| --- \| | \|  \| \| --- \| | \|  \| \| --- \| | \|  \| \| --- \| | \|  \| \| --- \| |  | 11 |
| **1.3** | Goal setting (outcome) | \|  \| \| --- \| | \|  \| \| --- \| | \|  \| \| --- \| | \|  \| \| --- \| |  | \|  \| \| --- \| | \|  \| \| --- \| |  |  | \|  \| \| --- \| |  |  | \|  \| \| --- \| | 8 |
| **3.1** | Social support (unspecified) | \|  \| \| --- \| |  | \|  \| \| --- \| | \|  \| \| --- \| |  |  |  |  | \|  \| \| --- \| | \|  \| \| --- \| | \|  \| \| --- \| | \|  \| \| --- \| | \|  \| \| --- \| | 8 |
| **2.2** | Feedback on behaviour | \|  \| \| --- \| |  | \|  \| \| --- \| | \|  \| \| --- \| |  |  | \|  \| \| --- \| |  | \|  \| \| --- \| |  | \|  \| \| --- \| |  |  | 6 |
| **2.3** | Self-monitoring of behaviour | \|  \| \| --- \| | \|  \| \| --- \| | \|  \| \| --- \| | \|  \| \| --- \| |  |  | \|  \| \| --- \| |  |  | \|  \| \| --- \| |  |  |  | 6 |
| **8.7** | Graded tasks |  | \|  \| \| --- \| | \|  \| \| --- \| | \|  \| \| --- \| | \|  \| \| --- \| | \|  \| \| --- \| | \|  \| \| --- \| |  |  |  |  |  |  | 6 |
| **9.1** | Credible source | \|  \| \| --- \| |  |  | \|  \| \| --- \| |  | \|  \| \| --- \| | \|  \| \| --- \| | \|  \| \| --- \| |  |  | \|  \| \| --- \| |  |  | 6 |
| **12.5** | Adding objects to the environment |  | \|  \| \| --- \| | \|  \| \| --- \| | \|  \| \| --- \| |  |  | \|  \| \| --- \| | \|  \| \| --- \| | \|  \| \| --- \| |  |  |  |  | 6 |
| **1.2** | Problem solving | \|  \| \| --- \| |  | \|  \| \| --- \| | \|  \| \| --- \| | \|  \| \| --- \| |  |  |  |  | \|  \| \| --- \| |  |  |  | 5 |
| **2.5** | Monitoring outcome(s) of behaviour by others without feedback | \|  \| \| --- \| | \|  \| \| --- \| |  | \|  \| \| --- \| |  |  |  | \|  \| \| --- \| |  |  |  |  | \|  \| \| --- \| | 5 |
| **6.1** | Demonstration of the behaviour | \|  \| \| --- \| |  | \|  \| \| --- \| | \|  \| \| --- \| |  |  | \|  \| \| --- \| |  |  |  | \|  \| \| --- \| |  |  | 5 |
| **8.1** | Behavioural practice/rehearsal | \|  \| \| --- \| |  |  | \|  \| \| --- \| |  |  | \|  \| \| --- \| |  |  |  | \|  \| \| --- \| |  |  | 4 |
| **2.4** | Self-monitoring of outcome(s) of behaviour |  | \|  \| \| --- \| | \|  \| \| --- \| | \|  \| \| --- \| |  |  |  |  |  |  |  |  |  | 3 |
| **2.7** | Feedback on outcome(s) of behaviour |  |  | \|  \| \| --- \| | \|  \| \| --- \| |  |  |  |  | \|  \| \| --- \| |  |  |  |  | 3 |
| **1.5** | Review behaviour goal(s) |  |  |  | \|  \| \| --- \| |  |  |  |  |  |  | \|  \| \| --- \| |  |  | 2 |
| **1.7** | Review outcome goal(s) |  |  |  | \|  \| \| --- \| |  |  |  |  | \|  \| \| --- \| |  |  |  |  | 2 |
| **2.1** | Monitoring of behaviour by others without feedback |  |  |  |  |  |  |  | \|  \| \| --- \| |  |  |  | \|  \| \| --- \| |  | 2 |
| **3.3** | Social support (emotional) |  |  | \|  \| \| --- \| |  | \|  \| \| --- \| |  |  |  |  |  |  |  |  | 2 |
| **5.1** | Information about health consequences |  |  |  | \|  \| \| --- \| | \|  \| \| --- \| |  |  |  |  |  |  |  |  | 2 |
| **7.1** | Prompts/cues |  |  | \|  \| \| --- \| | \|  \| \| --- \| |  |  |  |  |  |  |  |  |  | 2 |
| **10.3** | Non-specific reward |  |  | \|  \| \| --- \| | \|  \| \| --- \| |  |  |  |  |  |  |  |  |  | 2 |
| **10.9** | Self-reward |  |  | \|  \| \| --- \| | \|  \| \| --- \| |  |  |  |  |  |  |  |  |  | 2 |
| **12.1** | Restructuring the physical environment |  |  | \|  \| \| --- \| | \|  \| \| --- \| |  |  |  |  |  |  |  |  |  | 2 |
| **15.4** | Self-talk |  |  | \|  \| \| --- \| | \|  \| \| --- \| |  |  |  |  |  |  |  |  |  | 2 |
| **1.6** | Discrepancy between current behaviour and goal |  |  | \|  \| \| --- \| |  |  |  |  |  |  |  |  |  |  | 1 |
| **2.6** | Biofeedback |  |  |  | \|  \| \| --- \| |  |  |  |  |  |  |  |  |  | 1 |
| **3.2** | Social support (practical) |  |  |  | \|  \| \| --- \| |  |  |  |  |  |  |  |  |  | 1 |
| **6.2** | Social comparison |  |  |  | \|  \| \| --- \| |  |  |  |  |  |  |  |  |  | 1 |
| **8.2** | Behaviour substitution |  |  |  | \|  \| \| --- \| |  |  |  |  |  |  |  |  |  | 1 |
| **8.3** | Habit formation |  |  |  | \|  \| \| --- \| |  |  |  |  |  |  |  |  |  | 1 |
| **8.6** | Generalization of a target behaviour |  |  | \|  \| \| --- \| |  |  |  |  |  |  |  |  |  |  | 1 |
| **9.2** | Pros and cons |  |  |  | \|  \| \| --- \| |  |  |  |  |  |  |  |  |  | 1 |
| **10.2** | Material reward (behaviour) |  |  |  | \|  \| \| --- \| |  |  |  |  |  |  |  |  |  | 1 |
| **10.4** | Social reward |  |  |  | \|  \| \| --- \| |  |  |  |  |  |  |  |  |  | 1 |
| **10.6** | Non-specific incentive |  |  |  | \|  \| \| --- \| |  |  |  |  |  |  |  |  |  | 1 |
| **10.7** | Self-incentive |  |  |  | \|  \| \| --- \| |  |  |  |  |  |  |  |  |  | 1 |
| **11.2** | Reduce negative emotions |  |  |  | \|  \| \| --- \| |  |  |  |  |  |  |  |  |  | 1 |
| **12.2** | Restructuring the social environment |  |  |  | \|  \| \| --- \| |  |  |  |  |  |  |  |  |  | 1 |
| **12.3** | Avoidance/reducing exposure to cues for the behaviour |  |  |  | \|  \| \| --- \| |  |  |  |  |  |  |  |  |  | 1 |
| **13.1** | Identification of self as role model |  |  |  | \|  \| \| --- \| |  |  |  |  |  |  |  |  |  | 1 |
| **13.2** | Framing/reframing |  |  |  | \|  \| \| --- \| |  |  |  |  |  |  |  |  |  | 1 |
| **15.1** | Verbal persuasion about capability |  |  |  | \|  \| \| --- \| |  |  |  |  |  |  |  |  |  | 1 |
| **15.3** | Focus on past success |  |  |  | \|  \| \| --- \| |  |  |  |  |  |  |  |  |  | 1 |

**Key:** Studies are listed in alphabetical order (1) Agurs Collins et al., (2) Andrews et al., (3) Eakin et al., (4) Espeland et al., (5) Golhaber Fibert et al., (6) Kim et al 2006., (7) Kim et al 2014., (8) Koo et al., (9) Luley et al., (10) Mayer-Davis et al., (11) Schultz et al., (12) Vanninen et al., (13) Wolf et al.

**Additional file 1.14 Breakdown of frequency of BCTs used by Category for diet and physical activity behaviour**

| **BCT Category No.** | **BCTs by category** | **Total number of times this category of BCTs was used** | **Mean** | **Median** |
| --- | --- | --- | --- | --- |
| 1 | Goals and planning | 45 | 5 | 3 |
| 2 | Feedback and monitoring | 30 | 4.3 | 3 |
| 8 | Repetition and substitution | 17 | 2.43 | 2 |
| 12 | Antecedents | 15 | 2.5 | 2 |
| 3 | Social support | 13 | 4.33 | 2 |
| 4 | Shaping knowledge | 13 | 3.25 | 0 |
| 9 | Comparison of outcomes | 13 | 4.33 | 1 |
| 6 | Comparisons of behaviour | 9 | 3 | 2 |
| 10 | Reward and threat | 8 | 0.73 | 1 |
| 15 | Self-belief | 4 | 1 | 1 |
| 7 | Associations | 3 | 0.38 | 0 |
| 5 | Natural consequences | 2 | 0.33 | 0 |
| 13 | Identity | 2 | 0.4 | 0 |
| 11 | Regulation | 1 | 0.25 | 0 |
| 14 | Scheduled consequences | 0 | 0 | 0 |
| 16 | Covert learning | 0 | 0 | 0 |

**Additional file 1.15 Breakdown of BCTs ‘NOT’ used by category and individual BCTs**

| **Category No.** | **Category Label** | **BCT No.** | **BCT label** |
| --- | --- | --- | --- |
| 1 | Goals and planning | 1.8 | Behavioural contract |
|  |  | 1.9 | Commitment |
| 4 | Shaping knowledge | 4.2 | Information about antecedents |
|  |  | 4.3 | Re-attribution |
|  |  | 4.4 | Behavioural experiments |
| 5 | Natural consequences | 5.2 | Salience of consequences |
|  |  | 5.3 | Information about social and environmental consequences |
|  |  | 5.4 | Monitoring of emotional consequences |
|  |  | 5.5 | Anticipated regret |
|  |  | 5.6 | Information about emotional consequences |
| 6 | Comparisons of behaviour | 6.3 | Information about others' approval |
| 7 | Associations | 7.2 | Cue signaling reward |
|  |  | 7.3 | Reduce prompts/cues |
|  |  | 7.4 | Remove access to the reward |
|  |  | 7.6 | Satiation |
|  |  | 7.7 | Exposure |
|  |  | 7.8 | Associative learning |
| 8 | Repetition and substitution | 8.4 | Habit reversal |
|  |  | 8.5 | Overcorrection |
| 9 | Comparison of outcomes | 9.3 | Comparative imagining of future outcomes |
| 10 | Reward and threat | 10.1 | Material incentive (behaviour) |
|  |  | 10.5 | Social incentive |
|  |  | 10.8 | Incentive (outcome) |
|  |  | 10.10 | Reward (outcome) |
|  |  | 10.11 | Future punishment |
| 11 | Regulation | 11.1 | Pharmacological support |
|  |  | 11.3 | Conserving mental resources |
|  |  | 11.4 | Paradoxical instructions |
| 12 | Antecedents | 12.4 | Distraction |
|  |  | 12.6 | Body changes |
| 13 | Identity | 13.3 | Incompatible beliefs |
|  |  | 13.4 | Valued self-identity |
|  |  | 13.5 | Identity associated with changed behaviour |
| 14 | Scheduled consequences | 14.1 | Behaviour cost |
|  |  | 14.2 | Punishment |
|  |  | 14.3 | Remove reward |
|  |  | 14.4 | Reward approximation |
|  |  | 14.5 | Rewarding completion |
|  |  | 14.6 | Situation-specific reward |
|  |  | 14.7 | Reward incompatible behaviour |
|  |  | 14.8 | Reward alternative behaviour |
|  |  | 14.9 | Reduce reward frequency |
|  |  | 14.10 | Remove punishment |
| 15 | Self-belief | 15.2 | Mental rehearsal of successful performance |
| 16 | Covert learning | 16.1 | Imaginary punishment |
|  |  | 16.2 | Imaginary reward |
|  |  | 16.3 | Vicarious consequences |

Note: For categories 2 ‘Feedback and monitoring’ and 3 ‘Social support’, all BCTs were used in interventions included in this review.

**Additional file 1.16 Moderator analysis of HbA_1c_ effect sizes for dietary BCTs**

|  |  |  | **Effect size** | **95% CI** |  | **Effect size** | **95% CI** |  | **Subgroup analysis** | |  |
| --- | --- | --- | --- | --- | --- | --- | --- | --- | --- | --- | --- |
| **BCT**  **No.** | **BCTs** | **k present (absent)** | **Present** | **Lower limit** | **Upper limit** | **Absent** | **Lower limit** | **Upper limit** | **Q** | **P** | **Difference** |
| **6.1** | Demonstration of the behaviour | 5 (8) | -0.972 | -1.314 | -0.629 | -0.294 | -0.56 | -0.028 | 9.377 | 0.002 | -0.678 |
| **8.1** | Behavioural practice/rehearsal | 3 (10) | -1.052 | -1.705 | -0.398 | -0.475 | -0.779 | -0.17 | 2.459 | 0.117 | -0.577 |
| **4.1** | Instruction on how to perform a behaviour | 12 (1) | -0.606 | -0.829 | -0.383 | -0.067 | -0.717 | 0.583 | 2.36 | 0.124 | -0.539 |
| **2.3** | Self-monitoring of behaviour | 9 (4) | -0.612 | -0.894 | -0.329 | -0.453 | -0.846 | -0.06 | 0.414 | 0.52 | -0.159 |
| **12.3** | Avoidance/reducing exposure to cues for the behaviour | 4 (9) | -0.694 | -1.209 | -0.179 | -0.53 | -0.848 | -0.212 | 0.283 | 0.595 | -0.164 |
| **1.1** | Goal setting (behaviour) | 9 (4) | -0.603 | -0.878 | -0.328 | -0.46 | -0.855 | -0.065 | 0.339 | 0.56 | -0.143 |
| **1.5** | Review behaviour goal(s) | 3 (10) | -0.618 | -1.09 | -0.145 | -0.551 | -0.859 | -0.242 | 0.054 | 0.816 | -0.067 |
| **12.5** | Adding objects to the environment | 4 (9) | -0.612 | -1.033 | -0.191 | -0.542 | -0.851 | -0.234 | 0.068 | 0.794 | -0.07 |
| **2.2** | Feedback on behaviour | 6 (7) | -0.583 | -0.953 | -0.213 | -0.557 | -0.91 | -0.204 | 0.01 | 0.92 | -0.026 |
| **1.2** | Problem solving | 4 (9) | -0.557 | -1.051 | -0.064 | -0.583 | -0.906 | -0.26 | 0.007 | 0.932 | 0.026 |
| **1.7** | Review outcome goal(s) | 3 (10) | -0.536 | -0.943 | -0.129 | -0.573 | -0.861 | -0.284 | 0.021 | 0.884 | 0.037 |
| **2.7** | Feedback on outcome(s) of behaviour | 3 (10) | -0.53 | -0.977 | -0.082 | -0.585 | -0.888 | -0.282 | 0.04 | 0.841 | 0.055 |
| **1.4** | Action planning | 9 (4) | -0.525 | -0.778 | -0.272 | -0.639 | -1.081 | -0.198 | 0.194 | 0.659 | 0.114 |
| **3.1** | Social support (unspecified) | 8 (5) | -0.515 | -0.793 | -0.237 | -0.627 | -1 | -0.253 | 0.221 | 0.638 | 0.112 |
| **12.1** | Restructuring the physical environment | 3 (10) | 0.47 | -1.022 | 0.081 | -0.61 | -0.923 | -0.297 | -0.186 | 0.666 | 1.08 |
| **2.5** | Monitoring outcome(s) of behaviour by others without feedback | 4 (9) | -0.458 | -0.861 | -0.055 | -0.616 | -0.91 | -0.323 | 0.387 | 0.534 | 0.158 |
| **9.1** | Credible source | 11 (2) | -0.502 | -0.727 | -0.277 | -0.819 | -1.368 | -0.27 | 1.099 | 0.294 | 0.317 |
| **1.3** | Goal setting (outcome) | 10 (3) | -0.472 | -0.697 | -0.247 | -0.908 | -1.408 | -0.409 | 2.437 | 0.118 | 0.436 |
| **2.4** | Self-monitoring of outcome(s) of behaviour | 3 (10) | -0.251 | -0.633 | 0.131 | -0.714 | -0.99 | -0.438 | 3.71 | 0.054 | 0.463 |

**Additional file 1.17** **Moderator analysis of HbA_1c_ effect sizes for physical activity BCTs**

|  |  |  | **Effect size** | **95% CI** |  | **Effect size** | **95% CI** |  | **Subgroup analysis** | |  |
| --- | --- | --- | --- | --- | --- | --- | --- | --- | --- | --- | --- |
| **BCT**  **No.** | **BCTs** | **k present (absent)** | **Present** | **Lower limit** | **Upper limit** | **Absent** | **Lower limit** | **Upper limit** | **Q** | **P** | **Difference** |
| **4.1** | Instruction on how to perform a behaviour | 12 (1) | -0.608 | -0.837 | -0.379 | -0.13 | -0.754 | 0.494 | 1.983 | 0.159 | -0.478 |
| **9.1** | Credible source | 6 (7) | -0.762 | -1.124 | -0.401 | -0.398 | -0.73 | -0.066 | 2.12 | 0.145 | -0.364 |
| **8.1** | Behavioural practice/rehearsal | 4 (9) | -0.778 | -1.222 | -0.334 | -0.466 | -0.778 | -0.155 | 1.272 | 0.259 | -0.312 |
| **2.2** | Feedback on behaviour | 6 (7) | -0.667 | -0.976 | -0.358 | -0.426 | -0.763 | -0.088 | 1.071 | 0.301 | -0.241 |
| **6.1** | Demonstration of the behaviour | 5 (8) | -0.614 | -1.004 | -0.224 | -0.536 | -0.875 | -0.197 | 0.087 | 0.768 | -0.078 |
| **1.2** | Problem solving | 5 (8) | -0.647 | -1.111 | -0.183 | -0.539 | -0.869 | -0.208 | 0.139 | 0.709 | -0.108 |
| **8.7** | Graded tasks | 6 (7) | -0.568 | -0.881 | -0.254 | -0.547 | -0.885 | -0.208 | 0.008 | 0.928 | -0.021 |
| **2.3** | Self-monitoring of behaviour | 6 (7) | -0.531 | -0.852 | -0.21 | -0.586 | -0.912 | -0.259 | 0.055 | 0.815 | 0.055 |
| **1.3** | Goal setting (outcome) | 8 (5) | -0.539 | -0.811 | -0.266 | -0.591 | -0.987 | -0.195 | 0.046 | 0.831 | 0.052 |
| **12.5** | Adding objects to the environment | 6 (7) | -0.523 | -0.82 | -0.226 | -0.601 | -0.948 | -0.254 | 0.111 | 0.739 | 0.078 |
| **2.7** | Feedback on outcome(s) of behaviour | 3 (10) | -0.53 | -0.977 | -0.082 | -0.585 | -0.888 | -0.282 | 0.04 | 0.841 | 0.055 |
| **1.4** | Action planning | 11 (2) | -0.538 | -0.769 | -0.306 | -0.666 | -1.315 | -0.018 | 0.134 | 0.714 | 0.128 |
| **1.1** | Goal setting (behaviour) | 11 (2) | -0.53 | -0.772 | -0.289 | -0.654 | -1.17 | -0.138 | 0.182 | 0.67 | 0.124 |
| **2.5** | Monitoring outcome(s) of behaviour by others without feedback | 5 (8) | -0.44 | -0.818 | -0.061 | -0.639 | -0.942 | -0.336 | 0.647 | 0.421 | 0.199 |
| **3.1** | Social support (unspecified) | 8 (5) | -0.486 | -0.794 | -0.179 | -0.706 | -1.118 | -0.295 | 0.706 | 0.401 | 0.22 |
| **2.4** | Self-monitoring of outcome(s) of behaviour | 3 (10) | -0.251 | -0.633 | 0.131 | -0.714 | -0.99 | -0.438 | 3.71 | 0.054 | 0.463 |
